# Correction: Comparing cervical cerclage, pessary and vaginal progesterone for prevention of preterm birth in women with a short cervix (SuPPoRT): A multicentre randomised controlled trial

**DOI:** 10.1371/journal.pmed.1004594

**Published:** 2025-04-17

**Authors:** Natasha L. Hezelgrave, Natalie Suff, Paul Seed, Vicky Robinson, Jenny Carter, Helena Watson, Alexandra Ridout, Anna L. David, Susana Pereira, Fatemeh Hoveyda, Joanna Girling, Latha Vinayakarao, Rachel M. Tribe, Andrew H. Shennan, The SuPPoRT Collaborating Team

The following information is missing from the Funding Statement: This research was also supported by the Wellcome Clinical Research Career Development Fellowship (222970/Z/21/Z to NS).
