# Supplementary material for: Comparing cervical cerclage, pessary and vaginal progesterone for prevention of preterm birth in women with a short cervix (SuPPoRT): A multicentre randomised controlled trial
Source: PLoS Med. 2024 Jul 16;21(7):e1004427. doi: 10.1371/journal.pmed.1004427 (PMC11288449; doi:10.1371/journal.pmed.1004427)
Supplement: S1 Table — BMI, body mass index; sPTB, spontaneous preterm birth. Vaginal progesterone abbreviated to Progest. (DOCX) [file pmed.1004427.s001.docx]

S1 Table: Variables used for minimisation in the trial.

*BMI-body mass index, sPTB-spontaneous preterm birth. Vaginal progesterone abbreviated to Progest*

| Variable | | Intervention | | |
| --- | --- | --- | --- | --- |
|  |  | Cervical cerclage n (%)  n=128 | Pessary n (%)  n=125 | Progest n (%)  n=132 |
| Gestation at recruitment | 14-18^+6^ weeks | 51 (39.8) | 46 (36.8) | 50 (37.9) |
|  | 19-23^+6^ weeks | 77 (60.2) | 79 (63.2) | 82 (62.1) |
| BMI | <30 | 108 (84.4) | 107 (85.6) | 109 (82.6) |
|  | >30 | 20 (15.6) | 18 (14.4) | 23 (17.4) |
| Risk factor | Previous sPTB | 53 (41.7) | 48 (38.4) | 50 (37.9) |
|  | Previous cervical surgery only | 54 (42.2) | 58 (46.4) | 65 (49.2 |
|  | Incidental finding of a short cervix | 20 (15.6) | 19 (15.2) | 17 (12.9) |
