# Supplementary material for: Comparing cervical cerclage, pessary and vaginal progesterone for prevention of preterm birth in women with a short cervix (SuPPoRT): A multicentre randomised controlled trial
Source: PLoS Med. 2024 Jul 16;21(7):e1004427. doi: 10.1371/journal.pmed.1004427 (PMC11288449; doi:10.1371/journal.pmed.1004427)
Supplement: S2 Table — CL, cervical length; fFN, fetal fibronectin; PTB, preterm birth; sPTB, spontaneous preterm birth. Vaginal Progsterone abbreviated to Progest. (DOCX) [file pmed.1004427.s002.docx]

S2 Table: Prespecified subgroup analyses based on baseline risk factors of randomised women. *CL-cervical length, fFN-fetal fibronectin, PTB-preterm birth, sPTB-spontaneous preterm birth. Vaginal Progesterone abbreviated to Progest.*

|  | Cerclage  % (n) | Pessary  % (n) | Progest  % (n) | P value for overall difference | Cerclage vs Pessary RD (CI) | Cerclage vs Progest RD (CI) | Pessary vs Progest RD (CI) |
| --- | --- | --- | --- | --- | --- | --- | --- |
| Previous sPTB or mid-trimester loss | | | | | | | |
| PTB  <37 weeks | 44.4  (24/54) | 41.6  (20/48) | 37.3  (19/51) | 0.8 | -4.1 (-17.5 to 9.3) | 4.2 (-7.9 to 16.4) | -8.3 (-20.9 to 4.4) |
| No prior history of sPTB or mid-trimester loss | | | | | | | |
| PTB  <37 weeks | 19.2 (14/73) | 24.3  (18/74) | 16.1 (13/81( | 0.4 | -0.05 (-0.18 to 0.08) | 0.03 (-0.09 to 0.15) | -0.08 (-0.21 to 0.04) |
| CL<15 mm at baseline | | | | | | | |
| PTB  <37 weeks | 43.8  (7/16) | 26.7  (4/15) | 63.6  (7/11) | 0.1 | 17.1 (-16.0 to 5.0) | -19.9 (-57.3 to 17.5) | 37.0 (0.8 to 73.1) |
| fFN>200 ng/ml at first visit | | | | | | | |
| PTB  <37 weeks | 0  (0/2) | 20  (1/5) | 33.3  (1/3) | 0.7 | -20  (-55.1 to 15.1) | -33.3  (-86.7 to 20.0) | 13.3 (-50.5 to 77.2) |
