# Supplementary material for: Comparing cervical cerclage, pessary and vaginal progesterone for prevention of preterm birth in women with a short cervix (SuPPoRT): A multicentre randomised controlled trial
Source: PLoS Med. 2024 Jul 16;21(7):e1004427. doi: 10.1371/journal.pmed.1004427 (PMC11288449; doi:10.1371/journal.pmed.1004427)
Supplement: S3 Table — APH, antepartum haemorrhage; CS, cesarean section; GDM, gestational diabetes mellitus; ICP, intrahepatic cholestasis of pregnancy; PPH, postpartum haemorrhage; SVD, spontaneous vaginal delivery. (DOCX) [file pmed.1004427.s003.docx]

S3 Table: Safety Outcomes for intention to treat analysis. *APH-antepartum haemorrhage, CS-caesarean section, GDM-gestational diabetes mellitus, ICP-intrahepatic cholestasis of pregnancy, PPH-postpartum haemorrhage, SVD-spontaneous vaginal delivery.*

|  | Cerclage | | Pesssary | | Progesterone | |
| --- | --- | --- | --- | --- | --- | --- |
|  | Overall n | % (n) or median (quartiles) | Overall n | % (n) or median (quartiles) | Overall n | % (n) or median (quartiles) |
| Pre-eclampsia | 127 | 2.4 (3) | 122 | 5.8 (7) | 130 | 1.5 (2) |
| APH | 127 | 7.1 (9) | 122 | 7.4 (9) | 130 | 3.1 (4) |
| Other maternal complication (GDM/ICP) | 127 | 11.1 (14) | 122 | 10.6 (13) | 130 | 13.1 (17) |
| Maternal chorioamnionitis | 109 | 5.6 (6) | 110 | 8.2 (9) | 109 | 7.3 (8) |
| Antenatal hospital admissions (nights; median, IQR) | 127 | 2 (1,3) | 122 | 3 (1,5) | 129 | 4 (1,7) |
| Labour onset   - Spontaneous - Induced - Pre-labour CS - Unknown | 128 | 60.2 (77)  20.3 (26)  18.8 (24)  0.8 (1) | 122 | 51.6 (63)  29.5 (36)  6.3 (23)  0 (0) | 130 | 50.8 (66)  28.5 (37)  20.8 (27)  0 (0) |
| Mode of delivery   - SVD - Assisted delivery - Caesarean section - Unknown | 128 | 57.0 (73)  11.7 (15)  30.5 (39)  0.8 (1) | 122 | 55.7 (68)  10.7 (13)  28.7 (35)  0 (0) | 131 | 50.4 (66)  16.0 (21)  32.8 (43)  0.8 (1) |
| Post-partum complications   - PPH >1L | 123 | 12.2 (15) | 120 | 11.7 (14) | 128 | 11.7 (15) |
| Birth outcomes; median (IQR)   - Birth weight (g) - Apgar at 1 minute - Apgar at 5 minutes - Apgar score <7 at 5 minutes - Length of postnatal hospital stay (maternal) - Length of hospital stay   (baby)   - Requirement for oxygen at 28 days postnatal % (n) | 127  115  114  114  124  81  128 | 3135 (2388, 3510)  9 (8,9)  10 (9,10)  10.5 (12)  2 (1,3)  3 (1,8)  1.6 (2) | 122  109  109  109  120  71  122 | 2968 (2460, 3240)  9 (9,9)  10 (9,10)  4.6 (5)  3 (1,4)  4 (2,11)  4.1 (5) | 130  121  121  121  129  72  132 | 3140 (2705, 3550)  9 (9,9)  10 (9,10)  5 (6)  2 (1,4)  2 (1,6.5)  2.3 (3) |
