# Supplementary material for: Comparing cervical cerclage, pessary and vaginal progesterone for prevention of preterm birth in women with a short cervix (SuPPoRT): A multicentre randomised controlled trial
Source: PLoS Med. 2024 Jul 16;21(7):e1004427. doi: 10.1371/journal.pmed.1004427 (PMC11288449; doi:10.1371/journal.pmed.1004427)
Supplement: S4 Table — (DOCX) [file pmed.1004427.s004.docx]

S4 Table: Primary and secondary outcomes (and their components) for women in the SuPPoRT study according to per-protocol analysis

|  | Cerclage  % (n/100) | Pessary  % (n/105) | Progest  % (n/120) | P value  for overall difference | Cerclage vs Pessary  RD (CI) | Cerclage vs Progest  RD (CI) | Progest  vs Pessary  RD (CI) |
| --- | --- | --- | --- | --- | --- | --- | --- |
| PTB < 37 weeks of gestation | 34. (34) | 32.35 (33) | 25.0 (30) | 0.3 | 1.6  (-11.3 to 14.6) | 9.0  (-3.1 to 21.1) | -7.3  (-19.3 to 4.6) |
| Adverse perinatal outcome | 10.0 (10) | 7.6 (8) | 12.5 (15) | 0.5 | 2.4  (-5.4 to 10.1) | -2.5  (-10.8 to 5.8) | 4.9  (-2.9 to 12.7) |
| PTB < 34 weeks | 21.0 (21) | 17.7 (18) | 16.7 (20) | 0.7 | 3.3  (-7.6 to 14.2) | 4.3  (-6.1 to 14.7) | -0.9  (-10.9 to 9.0) |
| PTB < 30 weeks | 12.0 (12) | 7.8 (8) | 12.5 (15) | 0.5 | 4.2  (-4.1 to 12.4) | -0.5  (-9.2 to 8.2) | 4.7  (-3.2 to 12.5) |
| Time between intervention and delivery days (SD) | 113.3  (± 36.5) | 117.1  (± 34.6) | 117.9  (±39.1) | 0.6 | -388  (-1413 to 636) | -462  (-1418 to 524) | 74  (-905 to 1052) |
| Maternal Infection | 12.2  (12/98^a^) | 13.9  (14/101^a^) | 23.7  (28/118^a^) | 0.05 | -1.6  (-11.0 to 7.7) | -11.5  (-21.5 to -1.4) | 9.9  (-0.3 to 20.1) |
| IUGR (< 10^th^ Intergrowth centile) | 2.2  (2/93^a^) | 8.1  (8/99^a^) | 2.8  (3/109^a^) | 0.08 | -5.9  (-12.1 to 0.2) | -0.6  (-4.9 to 3.7) | -5.3 (  -11.5 to 0.9) |
| Baby in NNU at 28 days | 3.0 (3) | 6.9  (7/102^a^) | 5.8  (7) | 0.4 | -3.9 (-9.8 to 2.1) | -2.8 (-8.2 to 2.5) | -1.0 (-7.5 to 5.4) |
| Neonatal sepsis (by blood culture) | 1.0  (1/99^a^) | 2.0  (2/102^a^) | 2.6  (3/116^a^) | 0.7 | -1.0  (-4.3 to 2.4) | -1.6  (-5.1 to 1.9) | 0.6  (-3.3 to 4.6) |

*^a^different overall sample size due to missing data. BPD-bronchopulmonary dysplasia, HIE-hypoxic ischaemic encephalopathy, IVH-intraventricular haemorrhage, NEC-necrotising enterocolitis, NNU-neonatal unit, PTB-preterm birth, PVL-periventricular leukomalacia. Vaginal Progesterone abbreviated to Progest.*
