# Supplementary material for: Comparing cervical cerclage, pessary and vaginal progesterone for prevention of preterm birth in women with a short cervix (SuPPoRT): A multicentre randomised controlled trial
Source: PLoS Med. 2024 Jul 16;21(7):e1004427. doi: 10.1371/journal.pmed.1004427 (PMC11288449; doi:10.1371/journal.pmed.1004427)
Supplement: S5 Table — (DOCX) [file pmed.1004427.s005.docx]

S5 Table: Reported side effect profiles for women randomised to cerclage, pessary and vaginal progesterone

| Side effect | Severity | Cerclage  % (n/128) | Pessary  % (n/126) | Progesterone  % (n/132) | P value |
| --- | --- | --- | --- | --- | --- |
| Lower abdominal pain | Mild | 32.0 (41) | 33.3 (42) | 34.0 (45) | 0.94 |
|  | Severe | 3.1 (4) | 2.4 (3) | 9.8 (13) | 0.01 |
| Vaginal bleeding | Mild | 10.9 (14) | 16.6 (21) | 6.0 (8) | 0.03 |
|  | Severe | 0.8 (1) | 3.2 (4) | 0 (0) | 0.07 |
| Vaginal discomfort | Mild | 17.2 (22) | 24.6 (31) | 18.9 (25) | 0.31 |
|  | Severe | 1.6 (2) | 4.0 (5) | 6.8 (9) | 0.10 |
| Vaginal discharge | Mild | 38.3 (49) | 62.7 (79) | 50.8 (67) | 0.001 |
|  | Severe | 3.1 (4) | 16.7 (21) | 10.6 (14) | 0.002 |
| Difficulty voiding urine | Mild | 3.1 (4) | 6.3 (8) | 6.8 (9) | 0.36 |
|  | Severe | 1.6 (2) | 2.4 (3) | 0.8 (1) | 0.57 |
| Difficult defecation | Mild | 13.3 (17) | 23.0 (29) | 13.6 (18) | 0.06 |
|  | Severe | 0 (0) | 3.2 (4) | 2.3 (3) | 0.15 |
