# Supplementary figures and images for: Comparing cervical cerclage, pessary and vaginal progesterone for prevention of preterm birth in women with a short cervix (SuPPoRT): A multicentre randomised controlled trial
Source: PLoS Med. 2024 Jul 16;21(7):e1004427. doi: 10.1371/journal.pmed.1004427 (PMC11288449; doi:10.1371/journal.pmed.1004427)

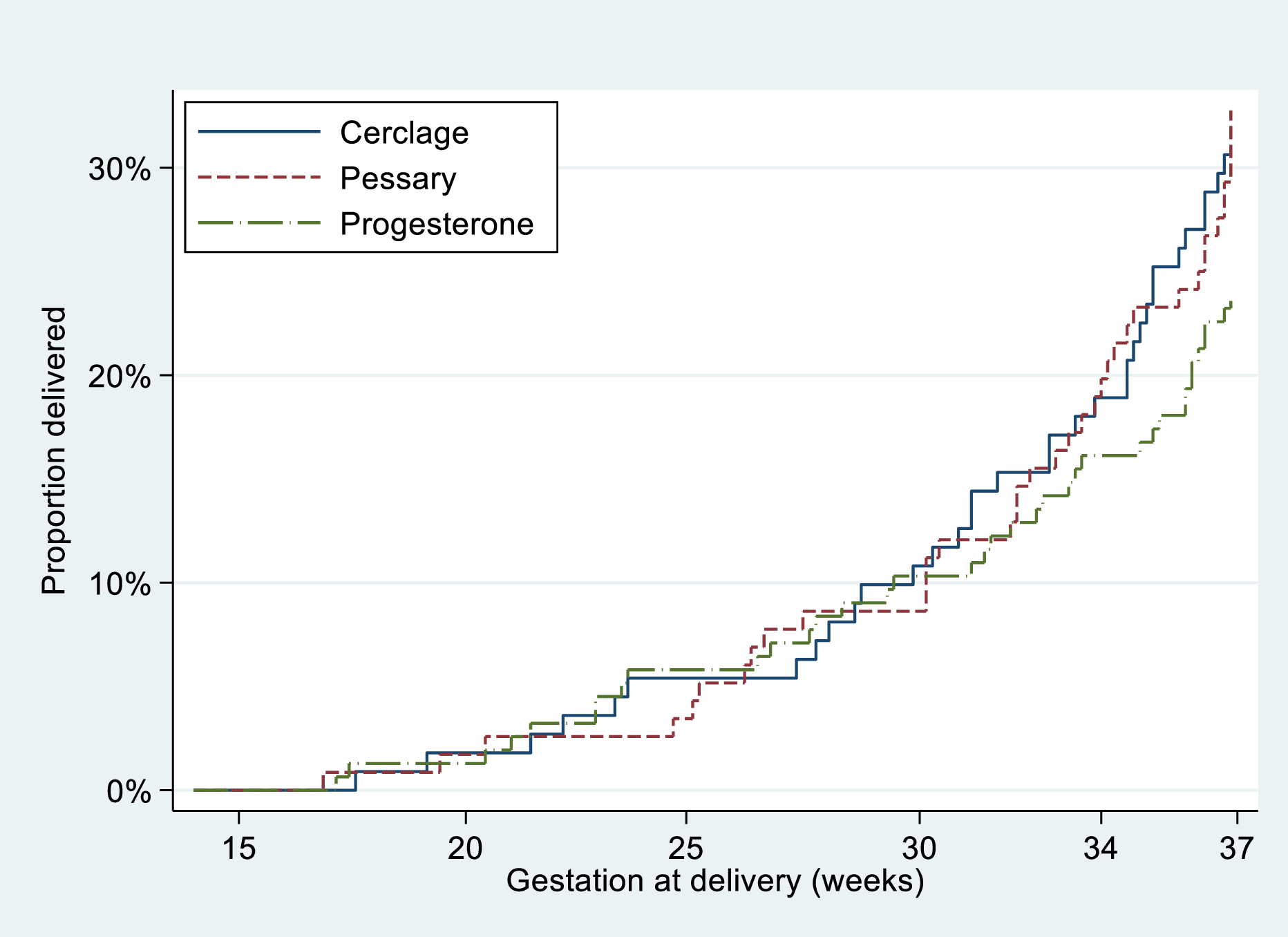

Supplement: S1 Fig — (TIF) [file pmed.1004427.s006.tif]
