# Supplementary material for: Comparing cervical cerclage, pessary and vaginal progesterone for prevention of preterm birth in women with a short cervix (SuPPoRT): A multicentre randomised controlled trial
Source: PLoS Med. 2024 Jul 16;21(7):e1004427. doi: 10.1371/journal.pmed.1004427 (PMC11288449; doi:10.1371/journal.pmed.1004427)
Supplement: S1 Statistical Analysis Plan — (DOCX) [file pmed.1004427.s009.docx]

**Support Study: Statistical analysis plan**

**SUPPORT: Stitch, progesterone or pessary: a randomised trial**

**The prevention of preterm birth in women who develop a short cervix**

**A multicentre randomised controlled trial to compare three treatments: cervical cerclage, cervical pessary and vaginal progesterone**

| Funder | National Institute for Health Research (NIHR) |
| --- | --- |
| Research ethics number | 15/LO/0485 |
| EudraCT number | 2015-000456-15 |
| ISRCTN number | ISRCTN13364447 |
| SAP version | 1.0 (19/4/2022) |
| Protocol version | 3.1 (23/10/2015) |

This statistical analysis plan (SAP) is intended to provide a detailed and comprehensive description of the planned methodology and analysis to be used in the SUPPORT randomised controlled trial.

**Trial summary and rationale**

In clinical practice, once a woman has been identified as being at risk of spontaneous preterm birth (sPTB), as defined by a short cervical length (<25 mm) on transvaginal ultrasound scan, a decision regarding prophylactic treatment must be made. There are three interventions with the potential to improve outcome in these high-risk women prior to 24 weeks’ gestation: cervical cerclage (stitch), vaginal progesterone treatment and cervical pessary. Each have been shown to have similar benefit in reduction of sPTB in women who develop a short cervix, but there have been *no* randomised control trials (RCTs) to compare the efficacy of each intervention; decision for intervention is currently based upon clinician experience and patient preference. This trial will evaluate whether a **cervical cerclage, cervical pessary** or **vaginal progesterone** are equally efficacious to prevent premature birth for women at high-risk of preterm birth who develop a short cervix (<25 mm) between 14+0 and 23+6 weeks’ gestation as measured by transvaginal ultrasonography.

**Aim and objectives:**

In a randomised controlled trial, to compare three evidence-based treatments for a short cervix detected by ultrasound scan in women at high risk of premature birth: cervical cerclage, cervical pessary and vaginal progesterone therapy.

The primary objectives of this study are:

- To determine if treatment with cervical cerclage, cervical pessary or vaginal progesterone in women at high risk of preterm birth who develop a short cervix by ultrasound measurement are equally efficacious to improve obstetric outcome by lengthening pregnancy and reducing the incidence of preterm delivery before 34 weeks’ gestation.
- To evaluate the impact of the interventions on short term neonatal outcomes, assessed as a composite of perinatal death (within 28 days) and major morbidity

The secondary objective is:

- To undertake an exploratory analysis to determine whether the response to intervention for a short cervix is related to the pre-intervention biomarker status (CVF, blood, saliva)

**Trial design**

An open label, multi-centre three-armed open label randomised controlled trial. Participants will be women with singleton pregnancies identified as high risk of sPTB (i.e., either a history of sPTB, second trimester loss, premature pre-labour fetal membrane rupture, previous cervical surgery or incidental findings of a short cervix) will be screened from 14 weeks’ gestation with transvaginal ultrasonography. Women who develop a short cervix will be randomised to one of 3 treatments: cervical cerclage (procedure to take place within 7 days of diagnosis, removed at 37 weeks’), cervical pessary (inserted at diagnosis and removed at 37 weeks’), vaginal progesterone (200 mg once daily per vagina until 34 weeks’ gestation from time of randomisation). At an appropriate time-point between time of randomisation and time of intervention, women will provide a saliva sample, CVF sample and blood sample (for biomarker analysis, optional, if facilities allow). These will be repeated every approximately every two weeks according to routine clinic visits thereafter until 28 weeks’ gestation.

**Primary Endpoint**:

- Delivery < 37 completed weeks’ gestation (powered).

**Secondary endpoints**

(Significance tests only for those with asterisks):

- Adverse perinatal outcome, defined as a composite outcome of death (antepartum/intrapartum stillbirths plus neonatal deaths prior to discharge from neonatal services) or one (or more) of intraventricular haemorrhage periventricular leukomalacia, hypoxic ischemic encephalopathy, necrotizing enterocolitis, bronchopulmonary dysplasia and sepsis. *
- Components of the composite outcome
- Delivery <30 & 34 completed weeks’ gestation *
- Gestation at delivery (medians)
- Time between intervention and delivery (Kaplan-Meier plots, censoring at 37 weeks).
- Requirement for Rescue Cerclage (bulging fetal membranes).
- Maternal morbidity
  - Infection at the time of delivery (any of maternal pyrexia/increased WCC or CRP during labour/positive blood culture/ clinical diagnosis of chorioamnionitis/received antibiotics for intrapartum infection) *
- Neonatal morbidity
  - IUGR (< 10^th^ Intergrowth centile)*
  - NNU at 28 days/discharge*
  - Neonatal sepsis (by blood culture) *
- Safety outcomes
  - Neonatal O_2_ at 28 days/discharge
  - Neonatal Apgar score at 1 and 5 min
  - Pre-eclampsia (clinical definition)
  - Intrahepatic cholestasis of pregnancy
  - Gestational diabetes mellitus
  - Antepartum haemorrhage
  - PPH (> 1,000ml)
  - Onset of labour (spontaneous/induced/ELCS)
  - Mode of delivery (SVD/assisted/caesarean section)
  - Antenatal hospital admissions
  - Median length of post-delivery hospital stay (maternal)
  - Median length of post-delivery neonatal hospital stay (neonatal)
- Compliance
  - Randomised treatment – ever received
  - Randomised treatment – stopped early
  - One or more of other interventions (cerclage, progesterone or pessary) added to randomised intervention
  - Crossover to other arm (cessation of randomised intervention)

**Randomisation**

At time of recruitment women are randomly assigned (1:1:1) to cerclage, progesterone or pessary. Randomisation is carried out online via the Medscinet web portal (www.medscinet.net). Due to the nature of the interventions, the study is not blinded to the clinician or patient. Recruiters and trial coordinators will not have access to the randomisation sequence. Women are informed at time of recruitment to which arm they have been randomised. A ‘minimisation’ procedure, using a computer-based algorithm, will be used to avoid chance imbalances in important stratification variables. Stratification variables will be a) gestation, b) BMI <30 or >30 kg/m2 c) risk factor (previous premature delivery <24 weeks & previous cervical surgery). Women do not know what treatment they will be allocated prior to recruitment. Medscinet wrote the randomisation program and hold the allocation code.

**Sample size calculation and framework**

From existing published evidence, we have good reason to believe that cerclage, vaginal progesterone and silicone pessary are all of approximately equal efficacy and reduce the rate of prematurity <37 weeks in women from 75% (untreated) to around 50% [6,8,9,15]. We therefore determine to confirm this by a 3-arm equivalence study. Equivalence is defined as agreement to within 20% (e.g.40% to 60%). We will declare equivalence only when we find a non-significant result for the primary endpoint. Following Jones *et al* (1996)18, we allow for differences in both directions in calculating the power. To allow for the multi-arm design, and to reduce the risk of false positive results, the exact confidence intervals will be considered only if the overall comparison shows evidence of a difference between the 3 arms of the study. Complete data on 170 women per arm (510 in all) will give us 95% power to detect difference clinically important differences of 20% or more in either direction. To allow for possible loss to follow-up of up to 5.5%, we aim to recruit 540 women in total.

| **Power** | **Difference** | **Numbers** |
| --- | --- | --- |
| 95% | 20% | 510 (170 per group) |
| 80% | 20% | 321 (107) |
| 90% | 20% | 420 |
| 90% | 22% | 348 |
| 90% | 24% | 294 |

**Statistical Analysis**

Primary analysis will be the intention to treat (ITT) population, defined as the subjects registered in the study at the point of randomization, regardless of the treatment received, excluding only participants who withdrew their consent.

A secondary analysis of the ‘per protocol ‘ (PP) population will also be carried out: limited to participants who were randomised, and who received only one intervention, according to the intervention administered, even if it was not the intended one. Women who received more than one intervention or none at all will be excluded from this analysis. Rescue cerclage for bulging fetal membranes will not be considered an additional intervention for this analysis however, as this is a secondary end-point in itself, and is not considered equivalent to cervical cerclage for a short cervix.

Baseline demographic, clinical and procedural data will be summarised using descriptive summary statistics, with results reported as numbers (percentages), means (SD) or medians (IQR), respectively. All data collected will be summarised overall, and by treatment allocation (ITT population).

The following null hypothesis will be tested: “There is no difference in the rate of sPTB <37 weeks gestation between the three arms of the study (cerclage, progesterone, Arabin pessary)”. All pairwise comparisons will be considered (A vs B, A vs C, B Vs C). The primary outcome (sPTB <37 weeks) will be compared between treatment groups using binary regression with an appropriate link, adjusting for possible differences in treatment efficacy between the study centre using robust standard errors (Rogers 1993). series of fixed effects. Results will be presented as both risk ratios (log link) and risk differences (identity link), leading to number needed to treat (NNT) if appropriate, according to CONSORT guidelines. We will not make a Bonferonni-type correction for multiple comparisons. Instead, we will carry out an overall test, for any difference between the 3 arms. As we are powering for equivalence in the maternal outcome, it is not anticipated that the neonatal outcomes will be different. However we will collect data on composite adverse perinatal outcome (not specifically powered for equivalence). Secondary outcomes will be compared between the treatment groups using binary regression for categorical variables, and linear regression for continuous variables, with study centre as a random effect. Time between intervention and delivery will be analysed using Kaplan Meier survival analysis. The primary and secondary analysis will be repeated on the PP population.

Given that high risk women with a history of invasive cervical surgery, and those with incidental findings of a short cervix may have a different pathophysiology to those women with a history of preterm birth, sub-group analysis will be performed according to history of previous preterm birth/late miscarriage or not. If numbers allow, sub-group analysis will also be performed by extending the main regression models to include cervical length of above and below 15mm, and pre-intervention quantitative fetal fibronectin concentration (<200ng/ml and >200 ng/ml).

Adverse events will be compared between study arms. Reported side-effects of treatment (lower abdominal pain, vaginal discharge, vaginal bleeding, vaginal discomfort, difficulty voiding, difficulty with defecation, and any others reported) will also be compared between treatment arms, as well as the proportion of women in each arm who did not receive the primary intervention (and reason for this), who stopped the primary intervention early (and reason for this), and who received alternative treatment (and reason for this).

Analysis will be performed using Stata version 17.1 or later. A two-sided p value of 0.05 will be used to determine statistical significance. Significance tests for any difference between the arms with be considered before examination of two-way comparisons. Results will be reported according to the CONSORT guidelines, bearing in mind the extensions for both multi-arm designs and equivalence trials (Moher 2010, Piaggio 2012, Jusczak 2013).

References

Edmund Juszczak, Douglas G Altman, Sally Hopewell, Kenneth Schulz. Reporting of Multi-Arm Parallel-Group Randomized Trials. Extension of the CONSORT 2010 Statement. JAMA. 2019;321(16):1610-1620. doi:10.1001/jama.2019.3087

Gilda Piaggio, Diana R Elbourne, Stuart Pocock, Stephen JW Evans, Douglas G Altman for the CONSORT Group. Reporting of Noninferiority and Equivalence Randomized Trials. Extension of the CONSORT 2010 Statement. JAMA. 2012;308(24):2594-2604

Moher D, Hopewell S, Schulz KF, et al. CONSORT 2010 explanation and elaboration: updated guidelines for reporting parallel group randomised trials. BMJ. 2010; 340:c869.

Rogers, W. H. 1993. sg17: Regression standard errors in clustered samples. Stata Technical Bulletin 13: 19–23. Reprinted in Stata Technical Bulletin Reprints, vol. 3, pp. 88–94. College Station, TX: Stata Press.

Planned tables and figures

Table 1: Baseline participant characteristics

|  | Cerclage | | Progesterone | | Arabin pessary | |
| --- | --- | --- | --- | --- | --- | --- |
|  | n | N(%) or mean (SD) | n | N(%) or mean (SD) | n | N(%) or mean (SD) |
| Age |  |  |  |  |  |  |
| BMI |  |  |  |  |  |  |
| Ethnicity  categories |  |  |  |  |  |  |
| Domestic violence |  |  |  |  |  |  |
| Drug use |  |  |  |  |  |  |
| Smoking  Current  Stopped prior to pregnancy   - Stopped during pregnancy - Ex-smoker - Never smoked |  |  |  |  |  |  |
| Any previous pregnancy |  |  |  |  |  |  |
| Risk factor at booking   - Previous preterm/PPROM - Previous late miscarriage - Previous cervical surgery - Incidental finding of short cervix |  |  |  |  |  |  |
| Previous spontaneous preterm birth/PPROM/late miscarriage |  |  |  |  |  |  |
| Previous cervical surgery only |  |  |  |  |  |  |
| Cervical length  15-25mm  <15mm |  |  |  |  |  |  |
| Mean gestation of intervention |  |  |  |  |  |  |

Table 2: Primary and secondary outcomes (and their components) for women in the SUPPORT study according to randomised intervention

|  | Cerclage | Arabin | Progesterone | P value for  overall between groups difference | Cerclage vs Arabin RD (p value) | Cerclage vs Progesterone (RD) (p value) | Progesterone vs Arabin RD (p value) |
| --- | --- | --- | --- | --- | --- | --- | --- |
| sPTB <37 weeks of gestation | x/x (x%) |  |  |  |  |  |  |
| Adverse perinatal outcome |  |  |  |  |  |  |  |
| Components of the adverse perinatal outcome |  |  |  |  |  |  |  |
| sPTB <34 weeks |  |  |  |  |  |  |  |
| sPTB<30 weeks |  |  |  |  |  |  |  |
| Time between intervention and delivery |  |  |  |  |  |  |  |
| Bulging fetal membranes and requirement for emergency cerclage |  |  |  |  |  |  |  |
| Maternal infection |  |  |  |  |  |  |  |
| IUGR (< 10^th^ Intergrowth centile) |  |  |  |  |  |  |  |
| NNU at 28 days/discharge |  |  |  |  |  |  |  |
| Neonatal sepsis (by blood culture) |  |  |  |  |  |  |  |

Table 3:Primary and secondary outcomes (and their components) for women in the SUPPORT study according to per protocol treatment

|  | Cerclage | Arabin | Progesterone | Overall between groups difference | P value | Cerclage vs Arabin RD (p value) | Cerclage vs Progesterone (RD) (p value) | Progesterone vs Arabin RD (p value) |
| --- | --- | --- | --- | --- | --- | --- | --- | --- |
| sPTB <37 weeks of gestation | x/x (x%) |  |  |  |  |  |  |  |
| Adverse perinatal outcome |  |  |  |  |  |  |  |  |
| Components of the adverse perinatal outcome |  |  |  |  |  |  |  |  |
| sPTB <34 weeks |  |  |  |  |  |  |  |  |
| sPTB<30 weeks |  |  |  |  |  |  |  |  |
| Time between intervention and delivery |  |  |  |  |  |  |  |  |
| Bulging fetal membranes and requirement for emergency cerclage |  |  |  |  |  |  |  |  |
| Maternal infection |  |  |  |  |  |  |  |  |
| IUGR (< 10^th^ Intergrowth centile) |  |  |  |  |  |  |  |  |
| NNU at 28 days/discharge |  |  |  |  |  |  |  |  |
| Neonatal sepsis (by blood culture) |  |  |  |  |  |  |  |  |

Table 4 Pre-specified sub-analysis (Intention to treat population)

|  | Treatment effect | | | | | | | | | | | | | | |
| --- | --- | --- | --- | --- | --- | --- | --- | --- | --- | --- | --- | --- | --- | --- | --- |
|  | RD or mean difference,p value cerclage vs progesterone | N | RD or mean difference,p value cerclage vs progesterone | N | P(interaction) | RD or mean difference,p value cerclage vs arabin | N | RD or mean difference,p value cerclage vs arabin | N | P(interaction) | RD or mean difference,p value progesterone vs arabin | N | RD or mean difference,p value progesterone vs arabin | N | P(interaction) |
| **Previous history of sPTB/PPROM/late miscarriage** | **yes** | **yes** | **no** | no |  | **yes** | **yes** | **no** | no |  | **yes** | **yes** | **no** | no |  |
| Sptb <37 |  |  |  |  |  |  |  |  |  |  |  |  |  |  |  |
| sPTB <34 |  |  |  |  |  |  |  |  |  |  |  |  |  |  |  |
| sPTB <30 |  |  |  |  |  |  |  |  |  |  |  |  |  |  |  |
| Composite perinatal outcome |  |  |  |  |  |  |  |  |  |  |  |  |  |  |  |
| Time from intervention to delivery |  |  |  |  |  |  |  |  |  |  |  |  |  |  |  |
| Bulging fetal membranes and requirement for emergency cerclage |  |  |  |  |  |  |  |  |  |  |  |  |  |  |  |
| Maternal infection |  |  |  |  |  |  |  |  |  |  |  |  |  |  |  |
| IUGR (< 10^th^ Intergrowth centile) |  |  |  |  |  |  |  |  |  |  |  |  |  |  |  |
| NNU at 28 days/discharge |  |  |  |  |  |  |  |  |  |  |  |  |  |  |  |
| Neonatal sepsis (by blood culture) |  |  |  |  |  |  |  |  |  |  |  |  |  |  |  |
| **Cervical length at baseline <15mm** | **no** | **no** | **yes** | yes |  | **no** | **no** | **yes** | yes |  | **no** | **no** | **yes** | yes |  |
| Sptb <37 |  |  |  |  |  |  |  |  |  |  |  |  |  |  |  |
| sPTB <34 |  |  |  |  |  |  |  |  |  |  |  |  |  |  |  |
| sPTB <30 |  |  |  |  |  |  |  |  |  |  |  |  |  |  |  |
| Composite perinatal outcome |  |  |  |  |  |  |  |  |  |  |  |  |  |  |  |
| Time from intervention to delivery |  |  |  |  |  |  |  |  |  |  |  |  |  |  |  |
| Bulging fetal membranes and requirement for emergency cerclage |  |  |  |  |  |  |  |  |  |  |  |  |  |  |  |
| Maternal infection |  |  |  |  |  |  |  |  |  |  |  |  |  |  |  |
| IUGR (< 10^th^ Intergrowth centile) |  |  |  |  |  |  |  |  |  |  |  |  |  |  |  |
| NNU at 28 days/discharge |  |  |  |  |  |  |  |  |  |  |  |  |  |  |  |
| Neonatal sepsis (by blood culture) |  |  |  |  |  |  |  |  |  |  |  |  |  |  |  |
| **Pre-intervention CVF qfFN concentration <200ng/ml** | **yes** | **yes** | **no** | **no** |  | **yes** | **yes** | **no** | **no** |  | **yes** | **yes** | **no** | **no** |  |
| Sptb <37 |  |  |  |  |  |  |  |  |  |  |  |  |  |  |  |
| sPTB <34 |  |  |  |  |  |  |  |  |  |  |  |  |  |  |  |
| sPTB <30 |  |  |  |  |  |  |  |  |  |  |  |  |  |  |  |
| Composite perinatal outcome |  |  |  |  |  |  |  |  |  |  |  |  |  |  |  |
| Time from intervention to delivery |  |  |  |  |  |  |  |  |  |  |  |  |  |  |  |
| Bulging fetal membranes and requirement for emergency cerclage |  |  |  |  |  |  |  |  |  |  |  |  |  |  |  |
| Maternal infection |  |  |  |  |  |  |  |  |  |  |  |  |  |  |  |
| IUGR (< 10^th^ Intergrowth centile) |  |  |  |  |  |  |  |  |  |  |  |  |  |  |  |
| NNU at 28 days/discharge |  |  |  |  |  |  |  |  |  |  |  |  |  |  |  |
| Neonatal sepsis (by blood culture) |  |  |  |  |  |  |  |  |  |  |  |  |  |  |  |

Table 5 Safety Outcomes

|  | Cerclage | | progesterone | | pessary | |
| --- | --- | --- | --- | --- | --- | --- |
|  | N | N (%) or mean (SD) | N | N (%) or mean (SD) | N | N (%) or mean (SD) |
| Pre-eclampsia |  |  |  |  |  |  |
| APH |  |  |  |  |  |  |
| GDM |  |  |  |  |  |  |
| ICP |  |  |  |  |  |  |
| Antenatal hospital admissions |  |  |  |  |  |  |
| Labour onset   - Spontaneous - Induced - Pre-labour CS |  |  |  |  |  |  |
| Mode of delivery   - SVD - Assisted delivery - Caesarean section |  |  |  |  |  |  |
| Post-partum complications   - PPH <1L |  |  |  |  |  |  |
| Birth outcomes   - Birth weight - Apgar and 1 and 5 - Median length of hospital stay (maternal) - Median length of hospital stay - (baby) - Requirement for oxygen at 28 days postnatal |  |  |  |  |  |  |

Figure 2

Kaplan-Meier Survival analysis for 3 arms, gestation at delivery

Figure 1: Consort diagram of flow of participants through the trial

Participants with CL<25mm screened n=x

Analysed n=x

Lost to follow up n=x

Met an exclusion criteria n= x

Analysed n=x

Analysed n=x

Lost to follow up n=x

Lost to follow up n=x

Randomised n=x

Refused randomisation n=x

Met inclusion criteria n=x

Randomised to cervical pessary

- Received allocated intervention n=x
- Received alternative intervention as an alternative to planned intervention n=x
- Received alternative intervention as an adjunct to planned intervention n=x
- Consent withdrawn,use of data permitted n=x
- Consent withdrawn data withdrawn n=x

Randomised to vaginal progesterone

- Received allocated intervention n=x
- Received alternative intervention as an alternative to planned intervention n=x
- Received alternative intervention as an adjunct to planned intervention n=x
- Consent withdrawn,use of data permitted n=x
- Consent withdrawn data withdrawn n=x

Randomised to cervical cerclage

- Received allocated intervention n=x
- Received alternative intervention as an alternative to planned intervention n=x
- Received alternative intervention as an adjunct to planned intervention n=x
- Consent withdrawn,use of data permitted n=x
- Consent withdrawn data withdrawn n=x
