# Supplementary material for: Comparing cervical cerclage, pessary and vaginal progesterone for prevention of preterm birth in women with a short cervix (SuPPoRT): A multicentre randomised controlled trial
Source: PLoS Med. 2024 Jul 16;21(7):e1004427. doi: 10.1371/journal.pmed.1004427 (PMC11288449; doi:10.1371/journal.pmed.1004427)
Supplement: S1 Trial Protocols — (ZIP) [file pmed.1004427.s010.zip › SuPPoRT trial protocols/SUPPORT Protocol V4 correct version 15.03.2018.pdf]

Study Protocol

SuPPoRT: Stitch, Progesterone or Pessary: a Randomised Trial

The prevention of pre-term birth in women who develop a short cervix

A multi-centre randomised controlled trial to compare three treatments; cervical cerclage, cervical pessary and vaginal progesterone;

Co-sponsors	Kings College London/ Guys and St Thomas' NHS Foundation Trust<br><br>Jackie Pullen<br>King's Health partners Clinical Trials Office<br>16th Floor Guys Hospital, Great Maze Pond<br>London SE1 9RT<br>Telephone: 02071885732<br>Fax: 02071888330<br>Email: Jackie.pullen@kcl.ac.uk<br><br>Kate Blake<br>Guys & St Thomas' NHS Foundation Trust<br>R&D department<br>16th Floor, Tower Wing, Great Maze Pond Rd<br>London SE1 9RT<br>Telephone: 0207188573<br>Fax: 02071883472<br>Email: Kate.Blake@gstt.nhs.uk
Funder	National Institute for Health Research (NIHR)
Funding Reference Number	DRF-2013-06-171
Chief Investigator	Professor Andrew Shennan
Co-Investigators	Dr Rachel Tribe, Dr Natasha Hezelgrave
Research Ethics number	15/LO/0485
EudraCT Number	2015-000456-15
ISRCTN Number	ISRCTN13364447
Version number and date	Version 4 15/03/18
Study Coordinator	Dr Natasha Hezelgrave
Scientist	Dr Evonne Chin-Smith
Trial statistician	Mr Paul Seed
Version and date of protocol amendments	

Study Synopsis

Title of clinical trial	A multi-centre randomised controlled trial to compare three treatments; cervical cerclage, cervical pessary and vaginal progesterone
Short Title/Acronym	SuPPoRT: Stitch, Progesterone or Pessary: a Randomised Trial
Chief Investigator	Professor Andrew Shennan<br>Women's Health Academic Centre<br>Kings College 0207<br>188 3639<br>andrew.shennan@kcl.ac.uk
Medical condition under investigation	Short cervical length and spontaneous preterm birth
Purpose of clinical trial	This trial will evaluate whether a cervical cerclage, cervical pessary or vaginal progesterone are equally efficacious to prevent spontaneous premature birth for women at high-risk of spontaneous preterm birth who develop a short cervix between 14^{+0} and 23^{+6} weeks' gestation.
Trial Design	Multicentre Randomised controlled trial
Primary Endpoint	Preterm birth rate < 37 weeks gestation
Sample size	540
Summary of eligibility criteria	Women with singleton pregnancies at high risk of preterm birth who are found to have cervical length <25 mm on transvaginal ultrasound between 14^{+0} weeks' until 23^{+6} weeks' gestation
Investigational medicinal products	Cervical cerclage vs Progesterone 200 mg PV, vs Arabin cervical pessary
Maximum duration of treatment of a participant	28 weeks

Trial Flowchart

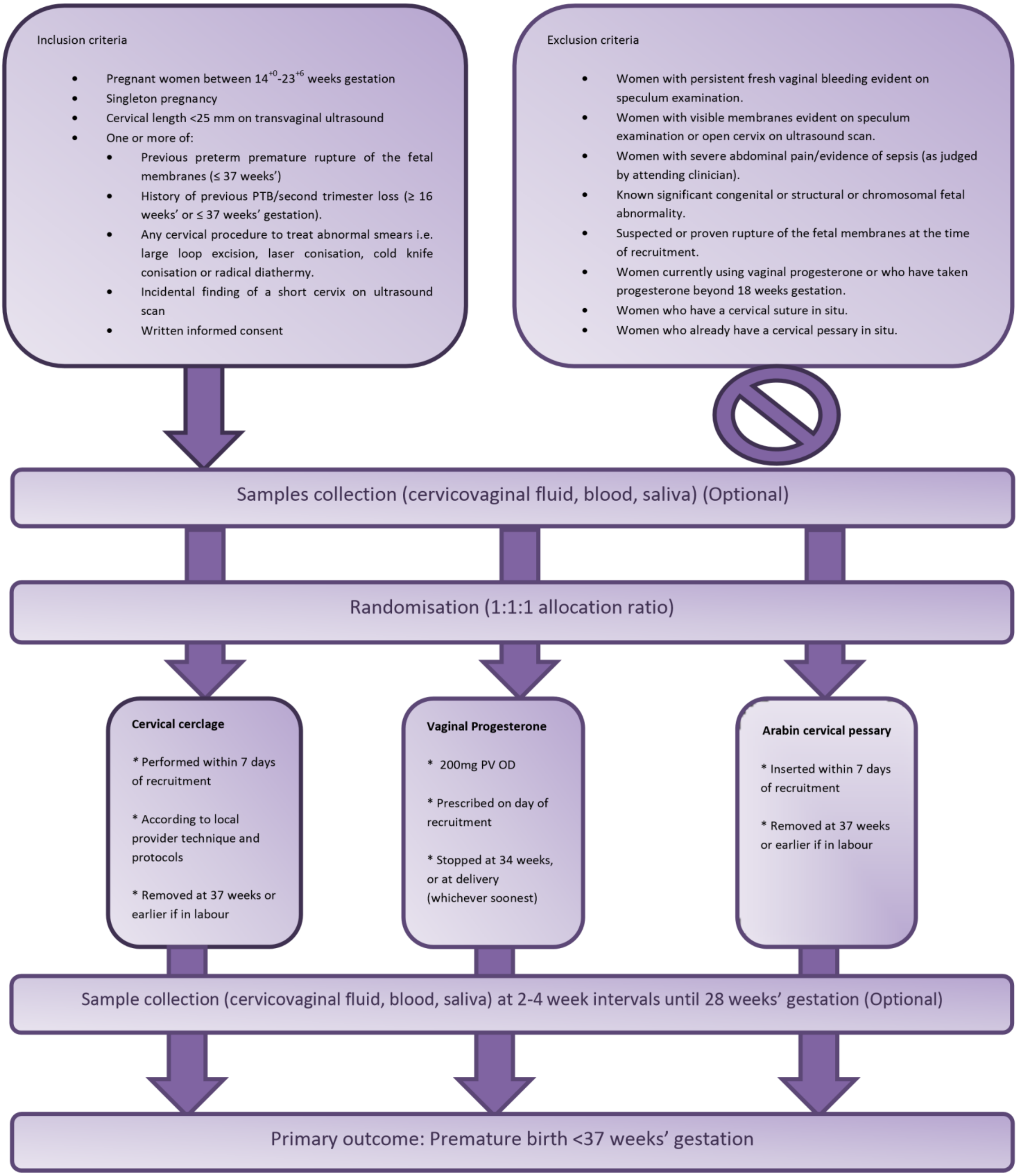

Contents

TABLE OF CONTENTS

STUDY SYNOPSIS 2

TRIALFLOWCHART 3

PROFESSIONAL SUMMARY 6

1 INTRODUCTION 7

1.1 Background 7

1.1.1 Cervical cerclage 7

1.1.2 Vaginal progesterone therapy 7

1.1.3 Cervical pessary 8

1.2 Prediction of premature birth using biomarkers 8

1.2.1 Fetal fibronectin 8

1.2.2 Natural antimicrobial peptides 8

1.2.3 Salivary progesterone 9

1.3 Rationale for study 9

1.4 Rationale for pre-intervention predictive biomarker testing 9

2. TRIAL AIM, DESIGN & STATISTICS 10

2.1 Aim 10

2.2 Objectives 10

2.3 Trial Design 10

2.4 Endpoints 11

2.4.1 Primary Endpoint 11

2.4.2 Secondary endpoints 11

2.5 Participants 11

2.5.1 Eligibility criteria 11

2.5.2 Exclusion criteria 12

2.5.3 Concomitant medication 12

3. RECRUITMENT 12

3.1 Identification of participants 12

3.2 Consent of participants 12

3.3 Study Visit 1: Randomisation and intervention 13

3.3.1 Cervical cerclage 13

3.3.2 Vaginal progesterone 13

3.3.3 Cervical pessary 14

3.3.4 Pre-intervention biomarker measurement 15

3.4 Subsequent study visits 15

3.5 Withdrawal from study 15

3.6 Expected duration of trial 16

3.7 Linkage of data 16

4. BIOLOGICAL SAMPLES 6

4.1 Sample collection and processing 16

4.1.1 Cervicovaginal fluid 16

4.1.2 Saliva sample 17

4.1.3 Blood samples 17

4.2 Storage 17

4.3 Analysis 17

5 SAMPLE SIZE, POWER AND STATISTICAL ANALYSIS 17

6 COLLECTION OF OUTCOME DATA 18

7 STUDY GOVERNANCE 18

7.1 Ethical Conduct of the study 18

7.2 Investigator responsibilities 18

7.3 Informed Consent 19

7.4 Study Site Staff 19

7.5 Data Recording 19

7.6 Data sharing and preservation strategy 19

7.7 GCP Training 19

7.8 Confidentiality 19

7.9 Data Protection 19

7.10 Study sponsor 19

7.11 NHS Trust Research and Development (R&D) 19

7.12 Study Coordinating Centre 20

7.13 Trial Steering Committee (TSC) 20

7.14 Data Monitoring Committee (DMC) 20

8 SAFETY 20
8.1 Assessment of Safety 20
8.2 Procedures for Recording and Reporting Adverse Events 20
8.2.1 Serious adverse Event (SAE), Serious Adverse Reaction (SAR) 20
8.2.2 Adverse events which do not require reporting 21
8.2.3 Reporting Responsibilities 22
8.2.4 Treatment stopping rules 22

9 PROTOCOL AMENDMENTS 22
10 PROTOCOL VIOLATIONS AND DEVAITIONS 22
11 DIRECT ACCESS TO SOURCE DATA AND DOCUMENTS 22
12 QUALITY ASSURANCES 22
13 DATAHANDLING 23
14 STUDY RECORD RETENTION 23
15 PUBLICATION POLICY 23
16 END OF THE STUDY 23
17 SCHEDULE OF VISITS 24
18 REFERENCES 25
19 APPENDIX 26

Professional Summary

Preterm birth, defined as birth before 37 weeks' completed gestation, is a major challenge facing modern obstetrics, with a global prevalence of 9.6% and over a million annual neonatal deaths[1]. The authors of the 2012 WHO report 'Born Too Soon: The Global Action Report on Preterm Birth, highlighted the unacceptably high and seemingly irretractable rate of prematurity worldwide, and emphasised the importance of continued research into biomarker strategies to predict women at risk and target clinical management appropriately[2] as well as research into optimal interventions to reduce the risk of preterm birth.

In clinical practice, once a woman has been identified as being at risk of spontaneous preterm birth (sPTB), as defined by a short cervical length (<25 mm) on transvaginal ultrasound scan, a decision regarding prophylactic treatment must be made. There are three interventions with the potential to improve outcome in these high-risk women prior to 24 weeks' gestation: cervical cerclage (stitch), vaginal progesterone treatment and cervical pessary. Each have been shown to have similar benefit in reduction of sPTB in women who develop a short cervix, but there have been *no* randomised control trials (RCTs) to compare the efficacy of each intervention; decision for intervention is currently based upon clinician experience and patient preference. This trial will evaluate whether a cervical cerclage, cervical pessary or vaginal progesterone are equally efficacious to prevent premature birth for women at high-risk of preterm birth who develop a short cervix (<25 mm) between 14^{+0} and 23^{+6} weeks' gestation as measured by transvaginal ultrasonography.

It will evaluate the impact of the three interventions on:

1. Obstetric outcome –premature birth rate <37 weeks' of gestation (primary), 34 weeks and 30 weeks (secondary outcomes).
2. Short-term neonatal outcomes –a composite of death and major morbidity

It will also explore whether success of the intervention can be predicted by pre-intervention biomarker and status (cervicovaginal fluid, blood and saliva).

Participants will be women with singleton pregnancies identified as high risk of sPTB (i.e. either a history of sPTB, second trimester loss, premature pre-labour fetal membrane rupture, previous cervical surgery or incidental findings of a short cervix) will be screened from 14 weeks' gestation with transvaginal ultrasonography. Asymptomatic women who develop a short cervix (<25 mm) will be randomised to either a cervical pessary (arabin), cervical cerclage or vaginal progesterone 200 mg once daily.

The trial is a UK multi-centre open label randomised controlled trial. The results of this study will be used to develop evidencebased local and national guidelines to better inform clinical practice.

1 INTRODUCTION

1.1 Background

There are 12.9 million premature births annually worldwide[1] and despite the magnitude of the problem, there is no established early pregnancy screening test or effective treatment for women once a high risk of spontaneous preterm birth (sPTB) is ascertained. The associated morbidity, mortality and high health costs are well documented. Only 39% of those infants born <26 weeks' gestation survive and 13% of these suffer severe cerebral palsy or sensory impairment[3]. Despite considerable efforts to introduce new therapies for the prevention and treatment of spontaneous preterm labour, sPTB rates are still rising; 6% of births in the UK are premature. sPTB is thought to be the result of multiple aetiologies influenced by a wide number of genetic, biological, psychosocial and environmental factors (e.g. multiple pregnancy, infection, placental abruption and stress) yet the chronology and aetiology of sPTB is insufficiently understood. Early sPTB is likely result from a complex interaction of maternal and/or fetal inflammatory responses which culminate in progressive cervical shortening and myometrial contractions.

A short cervix (detectable on transvaginal ultrasonography) has emerged as a useful predictor of sPTB in both low-risk and highrisk pregnancies. Risk of preterm birth is inversely related to cervical length; the shorter it is, the higher the risk of preterm birth. There may also be a number of other biomarkers [for example cervicovaginal fetal fibronectin (fFN) which can identify those women at the highest risk]. In clinical practice, once a woman has been identified as being at higher risk of sPTB by virtue of a short cervix, a decision regarding prophylactic treatment must be made. Three interventions have been proposed to treat patients with a short cervix; cervical cerclage[4-6], vaginal progesterone therapy[7, 8] and cervical pessary[9].

1.1.1 Cervical cerclage

This is the insertion of a 'purse string' suture around the cervix under regional anaesthesia. There is little consensus on the optimal procedure or technique (e.g. low/high vaginal, abdominal, tape/nylon, single/multiple, endocervical/purse string) or timing of insertion (elective, ultrasound indicated, pre conceptual). Furthermore, the mechanism of action is not understood; cerclage may offer a degree of structural support, but also plays a role in maintaining a biochemical barrier protecting membranes against exposure to ascending pathogens. It also is known to induce an inflammatory response which may encourage tissue repair. A 'history indicated' cerclage is inserted in early pregnancy (8-14 weeks' in women who have a history of late miscarriage of preterm birth. The largest randomised controlled trial comparing history-indicated cerclage with expectant management (n=1292), demonstrated that benefit of cerclage was only seen in women with three prior fetal losses/premature deliveries, where their risk of preterm birth reduced by more than half[10].

Ultrasound indicated cerclage involves the insertion of cerclage as a therapeutic measure following evidence of cervical length shortening (a predictor of preterm birth). Benefit of this cerclage has been reported in a subgroup of high risk women (history of preterm second-trimester loss or birth before 36 weeks of gestation) who have a cervix <25 mm in length, with meta-analysis[5] demonstrating a significant reduction in delivery before 35 weeks of gestation (relative risk [RR] 0.57; 95% CI 0.33–0.99 and RR 0.61; 95% CI 0.40–0.92) when compared with expectant management.

1.1.2 Vaginal progesterone therapy

Physiologically, progesterone is responsible for maintaining myometrial quiescence during pregnancy and supressing maternal immune system. As a result it has been advocated in the prevention of pre-term birth in singleton pregnancies. A Cochrane systematic review[7] revealed that prophylactic progesterone (intramuscular and vaginal administration of varying doses) was associated with a significant reduction in preterm birth <34 weeks (one study; 142 women; risk ratio (RR) 0.15; 95% confidence interval (CI) 0.04–0.64) and preterm birth at less than 37 weeks (four studies; 1255 women; RR 0.80; 95% CI 0.70–0.92). A metaanalysis of vaginal progesterone treatment in all women with a short cervix <25mm showed a reduction preterm birth (<33 weeks (RR, 0.58; 95% confidence interval [CI], 0.42 to 0.80), <35 weeks (RR, 0.69; 95% CI, 0.55 to 0.88), and <28 weeks (RR, 0.50; 95% CI, 0.30 to 0.81) and neonatal composite morbidity and mortality (RR 0.57; 95% CI, 0.40 – 0.81)[8]. The plasma (and/or uterine) concentration of progesterone required to reduce sPTB is unknown, and the mechanism of action uncertain. It is, however, frequently used in clinical practice (usual dose 200-400 mg).

The doses of vaginal progesterone used in completed trials are 100 mg (n=142)[11] or 200 mg (n=250).[12] Whilst vaginal progesterone use for the prevention of sPTB is unlicensed, it is commonly used in clinical practice in the UK and Europe and is also recommended for use in clinical practice in the USA by the Society for Maternal and Fetal Medicine. The Royal College of Obstetricians and Gynaecologists (RCOG) recommend vaginal progesterone use in the context of clinical trials to determine whether its use is associated with improved fetal, neonatal and/or infant outcome .

1.1.3 Cervical pessary

The Arabin pessary is a round and cone shaped flexible silicon device which is designed to be inserted into the vagina and sit in the upper vaginal fornix, to support and incline the cervix, with the intention to prevent premature cervical shortening and preterm birth. Goya *et al*[9] reported a multicentre randomised controlled trial (n=385) on pessary use in unselected women screened by TVS and showed that in women with a short cervical length (< 25 mm) between 18 and 22 weeks, the pessary reduced the rate of sPTB <34 weeks' gestation compared with controls (6% *vs* 27%, odds ratio 0·18, 95% CI 0·08 to 0·37; p<0·0001), with a significant difference detected in the occurrence of composite poor neonatal outcome. In a subsequent smaller RCT, 108 Asian women with a singleton pregnancy and a cervical length<25 mm at routine second-trimester TVS were randomized to a pessary and a control group. The mean gestational age at delivery was 38.1 weeks in the pessary group compared with 37.8 weeks in the expectant management group, with no significant differences in the rates of delivery before 28, 34 or 37 weeks[13].

1.2 Prediction of premature birth using biomarkers

1.2.1 Fetal fibronectin

Risk assessment and management of prematurity has been enhanced in recent years by the knowledge of pathophysiologically relevant biomarkers. fFN is an extracellular glycoprotein found in the decidua basalis in the pregnant uterus. Here it is concentrated between the decidua and the trophoblast and is best described as the 'glue' between the pregnancy and the uterus. In normal conditions fFN remains in this area, and very low concentrations are found in the cervico-vaginal secretions after 22 weeks. Higher concentrations at or after 22 weeks, or even as early as 13 weeks, detected by swab test in the cervicovaginal secretions have been associated with increased risk of sPTB[12]. The use of fFN for distinguishing preterm contractions from preterm labour (PTL) in symptomatic patients is now well accepted, but for screening asymptomatic patients deemed to be at high risk of sPTB its role has been less clear due to the limited sensitivity and positive predictive value of the standard qualitative test which provides a positive or negative result based on a threshold of 50 ng/ml. Goldenberg *et al* (1996) showed that the specificity of fFN was consistently 96-98%, which is valuable because it means women with a negative test can be safely discharged, but a sensitivity of 0.63 means an fFN test at 22-24 weeks predicts only just over half of sPTB at <28 weeks[14]. Studies using a quantitative assay (ELISA) demonstrate that the cervicovaginal fluid (CVF) quantitative fFN (qfFN) concentration correlates with the risk of sPTB and improved prediction has been demonstrated using thresholds both below and above 50 ng/ml (Abbott *et al*, In Press).

1.2.2 Natural antimicrobial peptides

More recently discovered biomarkers may represent alternative pathophysiological pathways and contribute to earlier risk prediction, with potential to increase the accuracy of existing tests. Our group has shown that the antimicrobial peptide (AMP) elafin (peptidase inhibitor 3 or skin-derived antiproteinase SKALP/Trappin-2) is a promising early predictive marker of cervical shortening and sPTB. This recent 'discovery' stage study determined that CVF elafin protein expression longitudinally in CVF from women at high risk of sPTB (previous history of late miscarriage or sPTB). The study demonstrated that in N=74 high risk women, CVF concentrations of elafin were significantly higher in women who developed a short cervix group (cases) compared to high risk women without cervical shortening (controls), regardless of gestation and treatment (ratio 2.71, CI 1.94 to 3.79, p<0.0005). Elafin concentrations predicted cervical shortening from 14 weeks' of pregnancy (n=11, ROC area = 1.00, p=0.0082) and remained three fold higher when cervical shortening was first detected (ratio 3.03 CI 1.92-4.81, p<0.0005). Elafin was unaltered by treatment (insertion of a cerclage or vaginal progesterone daily; ratio 1.28, CI: 0.88-1.87, p=0.196).

1.2.3 Salivary progesterone

A prospective study involving 90 high risk asymptomatic women in India showed that the mean value of salivary progesterone was significantly lower in all women who delivered at <37 weeks of gestation (n=38), compared with the term group (n=52; P < 0.001)[15]. Our research group have previously demonstrated a suppression of the normal gestational rise in salivary progesterone (24-34 weeks') in high risk women who delivered before 34 weeks' gestation (n=12), compared with those delivering spontaneously between 34 and 37 weeks' (n=28), and those delivering at term (n=64) (p=0.007 versus 34-37 weeks', and p=0.009 versus term deliveries). Analysis of progesterone concentrations in the saliva of >1000 high risk women is currently underway (The Poppy study).

1.3 Rationale for study

There have been no direct comparison of all three of these interventions and as such, there are no guidelines as to the optimal management of high-risk women who develop a sonographic short cervix. Women at high risk of sPTB who develop a short cervix cannot, at present, be counselled about which is the most suitable intervention to reduce their risk of delivering prematurely. Currently, cervical cerclage is the Royal College of Obstetricians (RCOG) recommended treatment for women with a short cervix <25mm and a history of 1 or more late miscarriages or preterm birth.

Our research group recently published an exploratory observational study[16] to evaluate the relationship between proinflammatory cytokines, cervical shortening and intervention, within which 37 women who developed a short cervix were randomised to treatment with vaginal progesterone (n=17) or cervical cerclage (n=19). A clinically important trend towards benefit (gestation at delivery) was noted in the cerclage group [mean gestation 33.7 weeks' cerclage versus 31.5 weeks' progesterone] although this result did not achieve statistical significance.

Alfirevic *et al*[17] compared retrospectively three separate cohorts of women with previous sPTB <34 weeks and short cervix treated with cerclage (*n*=142), vaginal progesterone (*n*=59) or a pessary (*n*=42). There were no significant differences in rates of perinatal loss, neonatal morbidity or sPTB, apart from a higher rate of sPTB before 34 weeks' gestation in the vaginal progesterone *vs* pessary groups. It was concluded that randomized comparisons of these three management strategies, or combinations thereof, are needed to determine the optimal management of these women. If a randomised study, such as the one proposed, showed that the three treatments were equally efficacious, then women and clinicians would have greater choice regarding treatment plans, expensive surgery (and potential complications) could be avoided. The pessary could be inserted at a later gestational age, when cerclage is no longer performed, potentially in an outpatient setting.

This randomised controlled trial will answer the current clinical dilemma of which is the most effective method to treat women at high risk of sPTB who develop a short cervix.

1.4 Rationale for pre-intervention predictive biomarker testing in parallel

Whilst sPTB culminates in a common pathway of myometrial activation, cervical dilation and rupture of membranes, it is initiated by a number of different factors, which hinders accurate identification of at risk women and application of a 'one size fits all' preventative intervention. It is anticipated that the multiple pathways and pathologies preceding cervical shortening and sPTB, and thus a woman's response to various preventative interventions, may be differentiated by the expression of different biomarkers. We predict that the observed intervention effects may be enhanced by selection of sub-groups of women according to their biomarker expression i.e. that the underlying pathophysiology may determine the success of one intervention over the other. 'Pre-intervention' samples of blood, saliva and CVF will be taken from all women when randomised, and two weekly thereafter until 24 weeks' (or delivery). Biomarkers measured will include fFN, natural antimicrobial peptides and other inflammation related markers and salivary progesterone, whilst creating a biobank of samples for future analysis of other potential candiate biomarkers. We will also analyse blood biomarkers and DNA extracted from whole blood for genetic differences (e.g. SNP polymorphisms) in women destined to deliver prematurely during pregnancy compared to those who deliver at term. Bacterial and viral DNA/RNA extracted from vaginal swabs to determine the relationship between candidate biomarkers the vaginal microbiome and response to intervention. ≥

2 Trial Aim, Objectives, Design and Statistics

2.1 Aim

In a randomised controlled trial, to compare three evidence-based treatments for a short cervix detected by ultrasound scan in women at high risk of premature birth: cervical cerclage, cervical pessary and vaginal progesterone therapy.

2.2 Objectives

The primary objectives of this study are:

- To determine if treatment with cervical cerclage, cervical pessary or vaginal progesterone in women at high risk of preterm birth who develop a short cervix by ultrasound measurement are equally efficacious to improve obstetric outcome by lengthening pregnancy and reducing the incidence of preterm delivery before 34 weeks' gestation.

- To evaluate the impact of the interventions on short term neonatal outcomes, assessed as a composite of perinatal death (within 28 days) and major morbidity

The secondary objectives are:

- To undertake an exploratory analysis to determine whether the response to intervention for a short cervix is related to the pre-intervention biomarker status (CVF, blood, saliva)

- To evaluate the acceptability to women and clinicians of each of the three treatment arms

- To assess the impact of both management strategies on health economic outcomes for mother and infant in terms of number of nights in each hospital setting; cost data to hospital discharge/28 days post natal (it is anticipated that a 6 month and 2 year follow up may be performed if funding is obtained).

2.3 Trial Design

An open label, multi-centre three armed randomised controlled trial. Women who develop a short cervix will be randomised to one of 3 treatments: cervical cerclage (procedure to take place within 7 days of diagnosis, removed at 37 weeks'), cervical pessary (inserted at diagnosis and removed at 37 weeks'), vaginal progesterone (200 mg once daily per vagina until 34 weeks' gestation from time of randomisation). At an appropriate time-point between time of randomisation and time of intervention, women will provide a saliva sample, CVF sample and blood sample (for biomarker analysis, optional, if facilities allow). These will be repeated every approximately every two weeks according to routine clinic visits thereafter until 28 weeks' gestation.

2.4 Endpoints

2.4.1 Primary Endpoint

i) Delivery < 37 completed weeks' gestation (powered).

2.4.2 Secondary endpoints

i) Adverse perinatal outcome, defined as a composite outcome of death (antepartum/intrapartum stillbirths plus neonatal deaths prior to discharge from neonatal services) or one (or more) of intraventricular hemorrhage,

periventricular leukomalacia, hypoxic ischemic encephalopathy, necrotizing enterocolitis, bronchopulmonary dysplasia and sepsis.

ii) Delivery <30 & 34 completed weeks' gestation.

iii) Gestation at delivery.

iv) Time between intervention and delivery.

v) Requirement for Rescue Cerclage (bulging fetal membranes).

vi) Other maternal and fetal outcomes: clinical course, therapies administered, maternal and fetal morbidity and mortality data until discharge or 28 days post-natal (whichever soonest).

vii) Participant and clinician's perceptions of treatment: questionnaires with a selection of participants at 0-2 weeks post procedure. Questionnaires at one year are planned if funding is obtained.

viii) Health costs at 28 days post-natal.

ix) Biochemical end-points (on available samples): cervical swabs will be taken to determine the presence of cervicovaginal infection and concentrations of biomarkers of preterm birth, infection and inflammation. Saliva samples will be collected for salivary hormone levels, and blood samples taken for inflammatory markers and genetic analysis. Results will be correlated with maternal and fetal outcomes.

2.5 Participants

2.5.1 Eligibility criteria

Women with singleton pregnancies who are found to have cervical length <25 mm on transvaginal ultrasound between 14^{+0} weeks' gestation (dated by ultrasound or LMP and adjusted for ultrasound estimated date of delivery once ultrasound performed if no miscarriage prior to dating ultrasound) until 23^{+6} weeks' gestation with written consent to participate and one or more of the following risk factors;

History of:

- Previous preterm premature rupture of the fetal membranes (≤ 37 weeks')
- History of previous PTB/second trimester loss (≥ 16 weeks' or ≤ 37 weeks' gestation).
- Any cervical procedure to treat abnormal smears i.e. large loop excision, laser conisation, cold knife conisation or radical diathermy.
- Incidental finding of a short cervix on ultrasound scan (e.g. at the time of anomaly scan).

2.5.2 Exclusion criteria

- Women with persistent fresh vaginal bleeding evident on speculum examination.
- Women with visible membranes evident on speculum examination or open cervix on ultrasound scan.
- Women with severe abdominal pain/evidence of sepsis (as judged by attending clinician).
- Known significant congenital or structural or chromosomal fetal abnormality.
- Suspected or proven rupture of the fetal membranes at the time of recruitment.
- Women currently using progesterone pessaries or who have taken progesterone, for the purpose of preventing preterm birth, beyond 14 weeks gestation.
- Women who have a cervical suture *in situ* (vaginal or abdominal)
- Women who already have a cervical pessary *in situ.*
- If the attending clinician feels that an individual woman is more suited to one treatment modality over another
- Insufficient understanding of the trial in the opinion of the Investigator
- Any contraindications or cautions to the investigational medicinal product including:
  - known allergy or hypersensitivity to progesterone.
  - hepatic dysfunction,
  - undiagnosed vaginal bleeding,

- mammary or genital tract carcinoma,
- thrombophlebitis,
- thromboembolic disorders, • cerebral haemorrhage,
- porphyria.

2.5.3 Concomitant medication

Participants will be permitted to use any concomitant medication (aside from the other study products themselves) required alongside the study drug/procedure/device. They will be enquired about and recorded at every visit. Any other medication or treatment that would form normal clinical management for these women at risk of preterm labour, i.e. antibiotics, corticosteroids, tocolytics, etc. will be permitted according to local hospital guidelines and clinician preference.

3 Recruitment

3.1 Identification of participants

Cervical length measurement is performed routinely at participating centres for women at high risk of preterm birth. It is measured using transvaginal ultrasound by a trained practitioner. Three measurements will be taken with a transvaginal probe (mm). Patient must have an empty bladder. The lowest of 3 measurements would be taken as final length (as per accepted clinical protocols).

High risk pregnant women who are found to have a cervical length of <25 mm during attendance at high risk surveillance antenatal clinic (hereafter referred to as prematurity surveillance clinic) will be identified. Their case notes will be reviewed for the patient's potential in to the trial and eligible patients will be informed of the study at time of diagnosis of short cervix. Members of the research team (midwives, doctors and scanning practitioners) will be familiar with the study so can discuss the research with women when required.

If a participant does not consent to sample collection (CVF, saliva, blood), or if the study site does not have the facilities to collect and process samples, this does not preclude trial entry. Eligible patients may be recruited from the ongoing NIHR funded Insight study (which includes biomarker measurement). If so, samples should only be collected once and can contribute to both studies. Valid consent will be obtained to permit data from samples can be shared between the Insight and Support databases.

3.2 Consent of participants

All participants will be provided with a written patient information leaflet with verbal translation available for non-English speaking women (via Language Line where available). Women will be consented by an appropriately trained (GCP) doctor. All women will be provided an opportunity to discuss the research with one of the research team if requested.

Women will be given information about the study and will be allowed adequate time (up to 48 hours, depending on length of cervix and urgency of treatment, as determined by the attending clinician) to read the patient information sheet and invited to consider participation. The original consent form will be kept in the Investigator Site File, a copy will be given to the participant to keep and a copy kept in the hospital notes. The patient's case records must be noted to show that they are participating in this trial.

3.3 Study visit 1: Randomisation and intervention (14+0-23+6 weeks' gestation)

At time of recruitment women will be randomly assigned (1:1:1) to cerclage, progesterone or pessary. Randomisation will be carried out online via the Medscinet web portal (www.medscinet.net). Users will be assigned a personal identifier number. Due

to the nature of the interventions, the study is not blinded to the clinician or patient. Recruiters and trial coordinators will not have access to the randomisation sequence. Women will be informed at time of recruitment to which arm they have been randomised. A 'minimisation' procedure, using a computer-based algorithm, will be used to avoid chance imbalances in important stratification variables. Stratification variables will be a) gestation, b) BMI <30 or >30 kg/m^2 c) risk factor (previous premature delivery <24 weeks & previous cervical surgery). Women will not know what treatment they will be allocated prior to recruitment. Medscinet will write the randomisation program and hold the allocation code. Contact information will be
obtained from the patient. Demographic measures will be entered into the central trial database.
Following randomisation, the obstetrician will then arrange for the intervention to be performed as the randomisation indicates. There is no 'emergency code break' procedure as the trial is an open label RCT.

3.4 Investigational medicinal products

It is unlikely that the participants will be affected by a serious adverse event or a serious adverse reaction in this trial. The medical and surgical interventions they will be receiving are those which would be offered routinely in clinical practice.

3.4.1 Cervical cerclage

The cerclage procedure will be booked at the time of recruitment. It will be performed (according to local practice and procedures) within 7 days of recruitment to the trial. A vaginal cerclage will be inserted in in the operating theatre by a clinician trained in the procedure, according to the technique preferred by the clinician. It is usually inserted under regional anaesthetic. Tocolysis, antibiotics and antenatal corticosteroids can be considered at the clinician's preference, but will be documented and considered in the analysis. The patient will usually go home the same day and will be followed up with two to four weekly transvaginal ultrasound scans (or more frequent if clinically indicated) according to local protocols. The suture is removed easily by exposing the cervix and cutting the knot, usually without the need for anaesthetic. This is done electively when a woman is 37 weeks' gestation, or if she presents in symptomatic preterm labour before labour becomes established, to avoid cervical trauma. The suture should also be removed if there is clinical evidence of chorioamnionitis. Cervical cerclage insertion is an established surgical procedure, which is associated with minimal risks. These include infection, miscarriage, bleeding, difficulty with suture removal and preterm prelabour rupture of membranes.

3.4.2 Vaginal progesterone

No significant adverse events have been reported with 200 mg vaginal progesterone. Vaginal progesterone once daily will be prescribed at the time of recruitment. Patients will be informed to insert vaginal progesterone every day until 34 weeks' gestation (or delivery, whichever is soonest). The patient will usually be followed up with two to four weekly transvaginal ultrasound scans or more frequently if clinically indicated) according to local protocols. If the cervix shortensand membranes are visible, prior to 24 weeks' gestation, a rescue cerclage will be inserted, according to local protocols. Vaginal progesterone will be stored as per manufacturers instructions and dispensed by the hospital pharmacy. Licensed indications are for adjunctive use with oestrogen in post-menopausal women with an intact uterus (HRT). Progesterone will not be used within its licensed indications for this study. Any preparation of vaginal progesterone (200 mg) currently used in clinical practicfe for the prevention of preterm birth, will be allowed for use in the trial (eg Cyclogest, Uterogestan) according to individual site clinical protocols.

Contraindications are:
• known allergy or hypersensitivity to progesterone.
• severe hepatic dysfunction,
• undiagnosed vaginal bleeding,
• mammary or genital tract carcinoma,
• thrombophlebitis,
• thromboembolic disorders, • cerebral haemorrhage,
• porphyria.

If unexplained, sudden or gradual, partial or complete loss of vision, proptosis or diplopia, papilloedema, retinal vascular lesions or migraine occurs during therapy, the drug should be discontinued and appropriate diagnostic and therapeutic measures instituted. Vaginal progesterone 200mg should be used cautiously in patients with conditions that might be aggravated by fluid retention (e.g. hypertension, cardiac disease, renal disease, epilepsy, migraine, asthma); in patients with a history of depression, diabetes, mild to moderate hepatic dysfunction, migraine or photosensitivity and in breast-feeding mothers. Any adverse events experienced should be recorded as per the AE/ SAE section 8 of this protocol.

Indications for stopping treatment are:

- Elective (preterm) delivery
- Fetal membrane rupture
- Symptomatic placenta praevia
- Sudden or gradual, partial or complete loss of vision,
- Proptosis or diplopia
- Papilloedema
- Other patient reaction or sensitivity to progesterone
- Patient request

No dosage adjustments are permitted. Women recruited will be at high risk of early delivery and therefore likely to be highly motivated to comply with treatment. We will monitor compliance carefully by asking local staff to review medication packs. Patients will be asked to return any unused medication at a 34 weeks' visit (or after delivery) whichever soonest, which will be recorded in the online database. If progesterone has been stopped for reasons other than those listed above it can be restarted at any time up to 34 weeks of gestation.

This trial is deemed by the Sponsor to be a Type A trial as the IMP is used routinely with no higher risk to the patient than standard medical care. All drugs will be supplied from pharmacy stock in standard packaging. No clinical trial labelling is required and no trial specific prescription is required unless stipulated by individual sites.

3.4.3 Cervical pessary

The product must be stored at room temperature. The appropriately sized (see appendix 1) pessary will be inserted within 7 days of recruitment by the attending clinician, who will be trained in the procedure. They will be given detailed written instructions about its subsequent management. It will be removed by a trained clinician at 37 weeks' gestation (or in the event of established labour). The patient will be usually be followed up with two to 4 weekly transvaginal ultrasound scans (or more frequent if clinically indicated) according to local protocols. If the cervix shortens and membranes become visible prior to 24 weeks' gestation, a rescue cerclage will be inserted, according to local protocols

Indications for stopping treatment include:

- Pessary dislodges or falls out twice or more
- Patient request

There might be some increase in vaginal discharge, however there is no evidence to suggest increased risk of infection or other complications.

3.5 Pre- and Post intervention biomarker measurement

At a convenient time between randomisation and intervention, the following procedures will take place in order to obtain biomarker levels (if study site facilities allow and the participant has consented to them):

a) One sterile speculum examination and approximately 4 cervico-vaginal swabs (high vaginal and/or endocervical). The speculum examination will last approximately 20-30 seconds (normally 15 seconds if only 1 swab is taken as per routine clinical care after 18 weeks'). qfFN will be performed from 18 weeks' gestation.
b) A saliva sample (5 ml) will be obtained.
c) Two blood samples will be taken (2 x 15 ml/1 tablespoon), one for genetic analysis; one for biomarker analysis.

CVF sampling will be repeated, if facilities allow, at each subsequent routine clinic visit until 28 weeks' gestation. Sample collection is not mandatory for trial participation. Some women may only want to provide samples at a single visit or only provide one or more of the samples listed.

3.6 Subsequent study visits

All participants will be seen in a follow up appointment, normally between 2 and 4 weeks post-intervention. Subsequently, all follow up visits will be at the discretion of the attending clinician. Each visit will be documented on the online database. A transvaginal ultrasound cervical length measurement should be performed, and results recorded on the trial database, at each visit. At each visit, information will be obtained on compliance, adverse events or pregnancy complications.

The final study visit will take place between 34^{+0} and 38^{+0}-weeks' gestation. Patients randomised to progesterone will be asked to return all unused medication and empty blister packs, in order to collect compliance information. Patients randomised to pessary will return at 37 weeks for removal. Patients randomised to cerclage will be seen between 34 and 37 weeks, and an appointment made for removal in the appropriate clinical area (usually hospital birth centre). If delivery has occurred prior to this time, then study staff will contact the participants, to arrange a follow up visit and collection of unused medication. Other than the baseline post intervention visit and final study visit, all other trial visits will be times to routine clinic attendances according to local clinical protocols and clinician practice. Interim study data will therefore only be collected if the patient attends the appropriate department for routine purposes.

Note review will take place 28 days after delivery to capture to collect maternal, fetal and health economic data up until 28 days postnatal. A contact/visit with the participant is not required.

3.7 Withdrawal from study

Participation in the study is voluntary. A patient has the right to discontinue drug/pessary or completely withdraw from the study at any time for any reason. The Investigator has the right to discontinue a patient taking drug/pessary at any time if it is deemed to be in the patient's best interest. If a participant elects to withdraw from the study no further samples will be collected. Identifiable data or tissue already collected with consent would be retained and used in the study if the women consents for it to be so. Consent would be sought to collect use the participant's delivery details (if delivery has not yet occurred). If a participant who has given informed consent loses capacity to consent, the participant would be withdrawn from the study. Identifiable data or tissue already collected with consent would be retained and used in the study. No further data or tissue would be collected or any other research procedures carried out on or in relation to the participant. If the participant is withdrawn due to a serious adverse event, the Principal Investigator will arrange for follow-up visits or telephone calls until the event has resolved or stabilised. However as the participants are pregnant women data will be collected routinely to outcome (i.e. delivery) and used in the analysis unless the consent to collect the outcome is specifically refused by the participant.

If an eligible patient is excluded or refuses to participate, the reasons for this will be documented where possible for input into trial metrics.

3.7 Expected duration of the trial

The end of the trial will be defined as 28 days post-delivery or discharge from hospital (whichever sooner) of the last recruited participant and infant. To achieve our projected numbers, we will involve at least three centres, each receiving referrals from satellite units, and will recruit over a 36 month period. Each site has a busy preterm birth surveillance clinic, with approximately 500 women screened each year. We anticipate that 30% of these (120/year) will develop a short cervix (< 25mm, based on our current EQUIPP database >20000 women at high risk of sPTB), and 50% (60) will be willing to be randomised in an RCT. Therefore over 36 months with recruitment from 3 centres, we would potentially be able to randomise 540 women with short cervixes.

3.8 Linkage of data

At the time of recruitment a unique study number will be allocated to the patient. Data will be recorded on a password protected KCL computer in order that recruits can be contacted and delivery outcomes recorded. The anonymised research record will not contain any patient identifiable data.
All records will be anonymised at time of data entry in accordance with the Data Protection Act 1998.

A bespoke internet based data management system has been designed and built by MedSciNet and maintained by them. Varying levels of access are available from simple user to global administrator, each level requiring individual log-in and password. Minimal identifiers will be used on the main study database (initials, date of birth and unique study number). Contact details to ensure data completeness will be stored on a separate database linked only to study ID. Data will be entered directly into the Preterm Birth Studies database, and data quality will be assured by regular data monitoring. Women will be followed up until postnatal discharge. Paper copies of consent forms will be stored numerically (by study ID) and kept in a secure location in accordance to the Data Protection act 1998.

4 Biological samples

4.1 Sample collection and processing

4.1.1 Cervicovaginal fluid

Participants will be asked to lie on a couch, having removed their underwear, with a sheet over their waist to maintain dignity. They will be aware that a speculum will be used in the collection of their routine clinical fFN swab. A chaperone will be present.

The speculum will be inserted with water or a pea-sized amount of KY jelly as lubricant (because of the quantitative nature of the test, lubrication should be minimal to avoid any contamination of the swab but allow easy insertion of the speculum).

Approximately 4 swabs will be taken for a single speculum. For a high vaginal sample (for biomarkers and microbiology samples), the swab will be inserted into the posterior fornix of the vagina and rotated 360°. It should be in the posterior fornix for 10 seconds, and then removed. For an endocervical sample, the swab or cytobrush will be taken from the cervical os and rotated 360 degrees for 10 seconds. The whole process will take up to 30 seconds. A qfFN sample will be taken from the posterior fornix, rotated for 10 seconds, and processed according to manufacturer's instructions using the Rapid 10Q qfFN analyser.

4.1.2 Saliva sample

Participants will be asked to rinse their mouth thoroughly with water 10 minutes prior to producing the sample. The start time will be noted, and a sample of at least 5 ml of saliva will be collected into a 30 ml universal specimen container over approximately 10 minutes. The sample will be kept on ice between each salivation.

4.1.3 Blood samples

A phlebotomist, midwife or doctor will take 15 ml of blood via venepuncture into one EDTA and SST tube.

4.2 Storage

Samples and data will be stored for 25 years. Biological samples will be preserved and stored in accordance with the Human Tissue Act (2004). Samples will be stored in -80□C freezers in a secure freezer corridor. These are regularly maintained via company maintenance contracts. All the –80□C freezers are alarmed, and this is transmitted to via text message to the mobile phones of designated research staff. Freezer temperatures are recorded online and can be monitored remotely via the internet. All samples will be barcoded. Barcodes will link to study ID number. Information will be limited to members of the research team. Samples must be assigned a unique code as soon as possible after acquisition. The code will be unique to the group and the coding system designed in such a way that a code cannot be used more than once. A link between the participant consent form and the uniquely assigned code will be maintained to facilitate identification of stored material from participants who subsequently revoke consent. The participant id will be added to the consent form, which is then stored securely.

4.3 Analysis

Biological samples of saliva, blood and cervico-vaginal secretions will be stored for future analysis of candidate and other biomarkers implicated in preterm birth. A variety of molecular and proteomic approaches will be used to determine biomarker status (e.g. inflammatory markers can be measured using a range of immunoassays, Western blot, mass spectrometry and novel aptamer arrays). Swabs taken for microbiome studies will be analyse using a combination of microarray techniques and PCR techniques. A range of genomic approaches will be used for genetic analyses (DNA, RNA).

5 Sample size, power and statistical analysis

Our previous experience (captured by a robust database of outcome data from >2000 women attending our prematurity clinic) indicates that approximately 50% of women with short cervices (<25 mm) treated with cerclage) deliver early <37 weeks. From existing published evidence, we have good reason to believe that cerclage, vaginal progesterone and silicone pessary are all of approximately equal efficacy and reduce the rate of prematurity in women from 75% (untreated) to around 50% [6,8,9,15]. We therefore determine to confirm this by a 3-arm equivalence study. Equivalence is defined as agreement to within 20% (e.g. 40% to 60%). Following Jones *et al* (1996)[18], we allow for differences in both directions in calculating the power. Complete data on 170 women per arm (510 in all) will give us 90% power to detect difference clinically important differences of 20% or more in either direction. To allow for dropouts, we aim to recruit 540 women in total.

Analysis will be according to intention to treat. The main outcome is delivery before 37 weeks'. Results will be presented as both odds ratios and risk differences, leading to number needed to treat (NNT) if appropriate, according to CONSORT guidelines. Given that high risk women with a history of invasive cervical surgery, and those with incidental findings of a short cervix may have a different pathophysiology to those women with a history of preterm birth, sub-group analysis will be performed according to risk factor. As we are powering for equivalence in the maternal outcome, it is not anticipated that the neonatal outcomes will be different however we will collect data on composite neonatal end point (not specifically powered for equivalence).

For biomarker analysis, results will be analysed at each time point in a cross sectional analysis, and on a case-control basis. We will express the overall usefulness of each marker for prediction of the primary outcome as a ROC area (Receiver Operating Characteristic), with 95% confidence interval and p-value. We will describe the performance of the most useful markers in terms of sensitivity, specificity and related measures for selected cut-points. We will use logistic regression in order to identify possible useful combinations of markers; where possible, we will use the repeated measurements to describe the change in test performance with gestation.

6 Collection of outcome data

Women will be followed up until postnatal discharge. Neonates will be followed up to discharge or 28 days (whichever is sooner). Prompts on the database will alert the research midwife/assistant when each trial participant reaches her delivery date. Birth registers and in-patient records will also be used to track hospital admissions and pregnancy outcomes. Outcome data (medical and economic) will be collected by review of obstetric handheld/electronic notes. The primary and secondary outcome data will be collected after discharge from neonatal care.

Measurement of outcome (medical and economic): will be collected via electronic patient records, and/or hand-held patient notes. If information is not available, e.g. if the delivery occurred elsewhere, the patient, patient's GP or other hospital will be contacted. Gestational age will be calculated using ultrasound estimated date of delivery predicted at the 12-15 week scan, or last menstrual delivery if miscarriage occurs prior to dating scan.

7 Study Governance

7.1 Ethical Conduct of the study

The trial will be conducted in compliance with the principles of the Declaration of Helsinki (1996), the principles of Good Clinical Practice (GCP) and in accordance with all applicable regulatory requirements including but not limited to the Research Governance Framework and the Medicines for Human Use (Clinical Trial) Regulations 2004, as amended in 2006 and any subsequent amendments.This protocol and related documents will be submitted for review to the Research Ethics Committee (REC), and to the Medicines and Healthcare products Regulatory Agency (MHRA) for Clinical Trial Authorisation. The Chief Investigator will submit a final report at conclusion of the trial to the KHP-CTO (on behalf of the Sponsor), the REC and the MHRA within the timelines defined in the Regulations. A favourable ethical opinion will be obtained from the appropriate REC, MHRA and local R&D approval will be obtained prior to commencement of the study. Written informed consent will be obtained and documented in the participants hand-held notes. A sticker will also be placed on the participant's notes.

7.2 Investigator responsibilities

The Investigator is responsible for the overall conduct of the study at the site and compliance with the protocol and any protocol amendments. In accordance with the principles of GCP, the following areas listed in this section are also the responsibility of the Investigator. Responsibilities may be delegated to an appropriate member of study site staff. Delegated tasks must be documented on a Delegation Log and signed by all those named on the list.

7.3 Informed Consent

The Investigator is responsible for ensuring informed consent is obtained before any protocol specific procedures are carried out. The decision of a participant to participate in clinical research is voluntary and should be based on a clear understanding of what is involved. Participants must receive adequate oral and written information – appropriate Participant Information and Informed Consent Forms will be provided. The oral explanation to the participant should be performed an Investigator, and must cover all the elements specified in the Participant Information Sheet/Informed Consent. The participant must be given every opportunity to clarify any points they do not understand and, if necessary, ask for more information. The participant must be given sufficient time to consider the information provided. It should be emphasised that the participant may withdraw their consent to participate at any time without loss of benefits to which they otherwise would be entitled. The participant should be informed and agree to their medical records being inspected by regulatory authorities but understand that their name will not be disclosed outside the hospital. The Investigator or delegated member of the trial team and the participant should sign and date the Informed Consent

Form(s) to confirm that consent has been obtained. The participant should receive a copy of this document and a copy filed in the Investigator Site File (ISF).

7.4 Study Site Staff

The Principal Investigator and site staff must be familiar with the protocol and the study requirements. It is the Investigator's responsibility to ensure that all staff assisting with the study are adequately informed about the protocol and their trial related duties and be GCP trained.

7.5 Data Recording

The Investigator is responsible for the quality of the data recorded in the electronic clinical research files.

7.6 Data sharing and preservation strategy

The Investigators will comply with the Kings College London (KCL) principles on data sharing and preservation. The consent form states that other researchers may wish to access (anonymised) data in future. The trial statistician will (in collaboration with the CI) manage access rights to the data set. Prospective new users must demonstrate compliance with legal, data protection and ethical guidelines before any data are released. We anticipate that anonymised SUPPORT data will be shared with other researchers to enable international prospective on prediction of preterm birth. The Co-sponsors or their delegates will ensure all other documents required by GCP are retained in a research Master File.

7.7 GCP Training

All study staff must hold evidence of appropriate GCP training or undergo GCP training. This should be updated every two years throughout the trial.

7.8 Confidentiality

All laboratory specimens, evaluation forms, reports, and other records must be identified in a manner designed to maintain participant confidentiality. All records must be kept in a secure storage area with limited access. Clinical information will not be released without the written permission of the participant, except as necessary for monitoring and auditing by the Co-Sponsors, their designee, Regulatory Authorities, or the REC. The Investigator and study site staff involved with this study may not disclose or use for any purpose other than performance of the study, any data, record, or other unpublished, confidential information disclosed to those individuals for the purpose of the study. Prior written agreement from the Co- Sponsors or their designee must be obtained for the disclosure of any said confidential information to other parties.

7.9 Data Protection

All Investigators and study site staff must comply with the requirements of the Data Protection Act 1998 with regard to the collection, storage, processing and disclosure of personal information and will uphold the Act's core principles. Computers used to collate the data will have limited access measures via user names and passwords. Published results will not contain any personal data that could allow identification of individual participants

7.10 Study sponsor

Kings College London/Guys and St Thomas' NHS Foundation trust are co-sponsors of the study.

7.11 NHS Trust Research and Development (R&D)

Individual sites will only start recruitment once they have received approval from their NHS Trust Research and Development
(R&D) office. Applications to R&D offices will be submitted through the NIHR Co-ordinated System for gaining NHS permission

7.12 Study Coordinating Centre

The trial coordinating centre will be Division of Women's Health, Kings College London, where the study coordinator will be based. The Division of Women's Health will be responsible for study data entry, statistical analysis, servicing both the Data Monitoring Committee (DMC) and the Trial Steering Committee (TSC) and, in collaboration with the Chief Investigator and Local Research Midwives/Nurses, for the day to day running of the study including recruitment of sites and training of staff.

7.13 Trial Steering Committee (TSC)

The role of the TSC is to provide the overall supervision of the study. The TSC should monitor the progress of the study and conduct and advise on its scientific credibility. The TSC will consider and act, as appropriate, upon the recommendations of the DMC and ultimately carries the responsibility for deciding whether the trial needs to be stopped on the grounds of safety or efficacy. The TSC will consist of an independent chair and at least two other independent members (not involved in study recruitment and not employed by any organisation directly involved in study conduct. A representative(s) from the Preterm Birth Patient Public Involvement group will be included. The first meeting will take place 1 month after trial start date; frequency will be decided at the first meeting.

7.14 Data Monitoring Committee (DMC)

A DMC independent of the applicants and TSC will review the progress of the trial at least annually and provide advice on the conduct of the trial to the TSC. The committee will periodically review study progress and outcomes. The timings and content of the DMC reviews will be detailed in a DMC charter which will be agreed at its first meeting. The DMC will meet 3 months after the trial start date; frequency of meeting will be decided at the first meeting.

8 Safety

8.1 Assessment of Safety

This trial is deemed by the Sponsor to be a Type A trial as the IMP is used routinely with no higher risk to the patient than standard medical care.

The data monitoring committee will be established to ensure the wellbeing of study participants. The committee will periodically review study progress and outcomes as well as reports of serious adverse events (SAEs). The DMC will, if appropriate, make recommendations regarding the continuance of the study or modification of the study protocol.

8.2 Procedures for Recording and Reporting Adverse Events

8.2.1 Serious adverse Event (SAE), Serious Adverse Reaction (SAR)

The Medicines for Human Use (Clinical Trials) Regulations 2004 and Amended Regulations 2006 gives the following definitions:

Adverse Event (AE): Any untoward medical occurrence in a subject to whom a medicinal product has been administered including occurrences which are not necessarily caused by or related to that product.

Adverse Reaction (AR): Any untoward and unintended response in a subject to an investigational medicinal product which is related to any dose administered to that subject.

Unexpected Adverse Reaction (UAR): An adverse reaction the nature and severity of which is not consistent with the information about the medicinal product in question set out in the Investigator's Brochure (IB) relating to the trial in question (for any other investigational product)

Serious adverse Event (SAE), Serious Adverse Reaction (SAR) or Unexpected Serious Adverse Reaction (SUSAR):

Any adverse event, adverse reaction or unexpected adverse reaction, respectively, that

- Results in death
- Is life-threatening;
- Required hospitalisation or prolongation of existing hospitalisation;
- Results in persistent or significant disability or incapacity - Consists of a congenital anomaly or birth defect.
- Is otherwise considered medically significant by the investigator.

All AEs and SAEs must be recorded from the time a participant is randomized to treatment until 30 days after stopping taking study drug and until pregnancy outcome (28 days after delivery). The Investigator should ask about the occurrence of AEs/SAEs at every visit during the study. Open-ended and non-leading verbal questioning of the participant should be used to enquire about AE/SAE occurrence. Participants should also be asked if they have been admitted to hospital, had any accidents, used any new medicines or changed concomitant medication regimens. If there is any doubt as to whether a clinical observation is an AE/SAE, the event should be recorded. Hospitalisations for treatment planned prior to randomisation and hospitalisation for elective treatment of a pre-existing condition will not be considered as an SAE. Complications occurring during such hospitalisation will be AE/SAEs.

Important Medical Events (IME): Events that may not be immediately life-threatening or result in death or hospitalisation but may jeopardise the patient or may require intervention to prevent one of the other outcomes listed in the definition above should also be considered serious.

8.2.2 Adverse events which do not require reporting

Expected SAEs are those events which are expected in the patient population or as a result of the routine care/treatment of a patient. The interventions they will be receiving are those which would be offered routinely in clinical practice. Cervical cerclage insertion is an established surgical procedure, which is associated with minimal risks. These include infection, miscarriage, bleeding, difficulty with suture removal and preterm prelabour rupture of membranes. The cervical pessary is not associated with known risk. Progesterone pessaries have risk of allergy. Symptoms of overdose may include somnolence, dizziness, euphoria or dysmenorrhoea (latter not applicable for pregnant women). Treatment is observation and, if necessary, symptomatic and supportive measures should be provided.

Serious adverse events/reactions which are unrelated to these clinical procedures will be reported as SAEs.

Events that are primary or secondary outcome measures are not considered to be SAEs and will be reported in the normal way, on the appropriate electronic case report form.

Maternal:

- Premature labour
- Premature rupture of membranes
- Chorioamnionitis

Infant:

- Perinatal death (unless unexpected in this population)
- Low birth weight
- Requirement for supplemental oxygen or ventilation support
- Complications of prematurity (eg IVH, NEC, encephalopathy, seizures, hypoglycaemia) unless unexpected in this population Admission of the baby to the neonatal unit

In addition the following common pregnancy complication events will not be considered SAEs: hospitalisation for pre-eclampsia or pregnancy induced hypertension, hospitalisation for symptoms of preterm labour (e.g. rupture of membranes, vaginal bleeding); hospitalisation for maternal discomfort; hospitalisation for rest; hospitalisation for observation or monitoring for which the woman is admitted for a period of less than 12 h; delivery complications such as caesarean section or postpartum haemorrhage.

8.2.3 Reporting Responsibilities

All SAEs, SARs and SUSARs (excepting those specified in this protocol as not requiring reporting) will be reported immediately (and certainly no later than 24hrs) by the Investigator to the KHP-CTO and CI for review in accordance with the current Pharmacovigilance Policy.The KHP-CTO will report SUSARs to the regulatory authorities (MHRA, competent authorities of other EEA (European Economic Area) states in which the trial is taking place. The Chief Investigator will report to the relevant ethics committee. Reporting timelines are as follows:

- SUSARs which are fatal or life-threatening must be reported not later than 7 days after the sponsor is first aware of the reaction. Any additional relevant information must be reported within a further 8 days.
- SUSARs that are not fatal or life-threatening must be reported within 15 days of the sponsor first becoming
- aware of the reaction.

The Chief Investigator and KHP-CTO (on behalf of the *co*-sponsors), will submit a Development Safety Update Report (DSUR) relating to this trial IMP, to the MHRA and REC annually.

8.2.4 Treatment stopping rules

The trial may be prematurely discontinued by the Sponsor, Chief Investigator or Regulatory Authority on the basis of new safety information or for other reasons given by the DMEC/TSC regulatory authority or ethics committee concerned. If the trial is prematurely discontinued, active participants will be informed and no further participant data will be collected. The Competent Authority and Research Ethics Committee will be informed within 15 days of the early termination of the trial.

9 Protocol amendments

Any changes in research activity, except those necessary to remove an apparent, immediate hazard to the participant, must be reviewed and approved by the Chief Investigator and the Co-Sponsors notified. Substantial amendments to the protocol must be submitted in writing to the appropriate REC, Regulatory Authority and local R&D for approval prior to participants being enrolled into an amended protocol.

10 Protocol violations and deviations

The Investigator should not implement any deviation from the protocol except where necessary to eliminate an immediate hazard to trial participants. In the event that an Investigator deviate deviation from the protocol, the nature of and reasons for the deviation should be recorded in the electronic clinical research files. If this necessitates a subsequent protocol amendment, this will be submitted to the REC, Regulatory Authority and local R&D for review and approval by the Chief Investigator.

11 Direct Access to Source Data and Documents

Meetings will be held on a regular basis by the research team to monitor and audit the conduct of the research and review aspects of the SuPPoRT study's progress. The Investigator(s) will permit trial-related monitoring, audits, REC review, and regulatory inspections by providing the Sponsor(s), Regulators and REC direct access to source data and other documents (e.g. patients' case sheets, blood test reports, X-ray reports, histology reports etc).

12 Quality Assurances

Monitoring of this trial will be to ensure compliance with Good Clinical Practice and scientific integrity will be managed and oversight retained, by the KHP-CTO Quality Team.

13. Data handling

The Chief Investigator will act as custodian for the trial data. Patient data will be anonymised. All anonymised data will be stored on a password protected computer.All trial data will be stored in line with the Medicines for Human Use (Clinical Trials) Amended Regulations 2006 and the Data Protection Act and archived in line with the Medicines for Human Use (Clinical Trials) Amended Regulations 2006 as defined in the Kings Health Partners Clinical Trials Office Archiving SOP.

14 Insurance / Indemnity

The Co sponsor's King's College London and Guy's and St Thomas NHS Foundation Trust will provide insurance and indemnity.

15 Financial Aspects

Funding to conduct the trial is provided by Tommy's Charity and the National Institute for Health Research (NIHR).

16 Publication Policy

It is intended that the results of the study will be reported and disseminated at international conferences and in peer-reviewed scientific journals

17 End of the study

The end of study declaration will be submitted to the relevant authorities following 28 days or discharge from hospital (whichever soonest) of the last woman recruited to the project. The end of the study will be reported to the REC and Regulatory Authority within 90 days, or 15 days if the study is terminated prematurely.

The Investigators have the right at any time to terminate the study for clinical or administrative reasons.

Signature

Chief Investigator

15/03/18
Date

Schedule of Visits

Procedure	Screening & randomisation 14^{+0}-23^{+6} weeks gestation	Intervention (day 0) 14^{+0}-23^{+6} weeks gestation	Baseline visit (day) 1-4 weeks after procedure	Follow up visits (if clinically indicated)	Final visit 34^{+0}-38^{+6} weeks' gestation	Note review (no visit required)
Visit window (± days)	-7 to 0 days	Day 0	7-28 days	Approximately 2-4 weekly	3	**Discharge from Hospital or 28 days postnatal**
Informed consent	C					
Medical history and concomitant medications	C		C	O	C	
Transvaginal ultrasound Scan	C		C	O		
Biological sample collection	O		O	O		
Adverse events (AEs) and changes to interventions			C	O	C	C
Count un-used medication if appropriate					C	
Pessary/Cerclage removal booked/performed at 37 weeks' if appropriate					C	

C: Compulsory
0: Optional

14 References

1. http://www.marchofdimes.org/materials/global-report-on-birth-defects-the-hidden-toll-of-dying-and-disabledchildren-executive-summary.pdf.
2. Howson CP KM, Lawn JE, eds. Born too soon: the global action report on preterm birth. 2012.
3. Costeloe K, Group EPS. EPICure: facts and figures: why preterm labour should be treated. BJOG: An International Journal of Obstetrics & Gynaecology 2006;**113**:10-12.
4. To MS, Alfirevic Z, Heath VC, et al. Cervical cerclage for prevention of preterm delivery in women with short cervix: randomised controlled trial. Lancet 2004;**363**(9424):1849-53.
5. Berghella V, Odibo AO, To MS, et al. Cerclage for short cervix on ultrasonography: meta-analysis of trials using individual patient-level data. Obstet Gynecol 2005;**106**(1):181-9.
6. Owen J, Hankins G, Iams JD, et al. Multicenter randomized trial of cerclage for preterm birth prevention in highrisk women with shortened midtrimester cervical length. Am J Obstet Gynecol 2009;**201**(4):375.e1-8.
7. Dodd JM, Jones L, Flenady V, et al. Prenatal administration of progesterone for preventing preterm birth in women considered to be at risk of preterm birth. The Cochrane database of systematic reviews 2013;**7**:Cd004947.
8. Romero R, Nicolaides K, Conde-Agudelo A, et al. Vaginal progesterone in women with an asymptomatic sonographic short cervix in the midtrimester decreases preterm delivery and neonatal morbidity: a systematic review and metaanalysis of individual patient data. Am J Obstet Gynecol 2012;**206**(2):124.e1-19.
9. Goya M, Pratcorona L, Merced C, et al. Cervical pessary in pregnant women with a short cervix (PECEP): an openlabel randomised controlled trial. The Lancet;**379**(9828):1800-06.
10. Final report of the Medical Research Council/Royal College of Obstetricians and Gynaecologists multicentre randomised trial of cervical cerclage. MRC/RCOG Working Party on Cervical Cerclage. British journal of obstetrics and gynaecology 1993;**100**(6):516-23.
11. da Fonseca EB, Bittar RE, Carvalho MH, et al. Prophylactic administration of progesterone by vaginal suppository to reduce the incidence of spontaneous preterm birth in women at increased risk: a randomized placebocontrolled double-blind study. American journal of obstetrics and gynecology 2003;**188**(2):419-24.
12. Fonseca EB, Celik E, Parra M, et al. Progesterone and the risk of preterm birth among women with a short cervix. New England Journal of Medicine 2007;**357**(5):462-69.
13. Hui SY, Chor CM, Lau TK, et al. Cerclage pessary for preventing preterm birth in women with a singleton pregnancy and a short cervix at 20 to 24 weeks: a randomized controlled trial. Am J Perinatol 2013;**30**(4):283-8.
14. Goldenberg RL, Mercer BM, Meis PJ, et al. The preterm prediction study: fetal fibronectin testing and spontaneous preterm birth. NICHD Maternal Fetal Medicine Units Network. Obstet Gynecol 1996;**87**(5 Pt 1):643-8.
15. Priya B, Mustafa MD, Guleria K, et al. Salivary progesterone as a biochemical marker to predict early preterm birth in asymptomatic high-risk women. BJOG : an international journal of obstetrics and gynaecology 2013;**120**(8):1003-11.
16. Chandiramani M, Seed PT, Orsi NM, et al. Limited relationship between cervico-vaginal fluid cytokine profiles and cervical shortening in women at high risk of spontaneous preterm birth. PloS one 2012;**7**(12):e52412.
17. Alfirevic Z, Owen J, Carreras Moratonas E, et al. Vaginal progesterone, cerclage or cervical pessary for preventing preterm birth in asymptomatic singleton pregnant women with a history of preterm birth and a sonographic short cervix. Ultrasound in Obstetrics & Gynecology 2013;**41**(2):146-51.
18. Jones B, Jarvis P, Lewis J, et al. Trials to assess equivalence: the importance of rigorous methods. Bmj 1996;**313**(7048):36-39.

Appendix: Sizing of Arabin pessary

Table 1 Proposed sizes of the Arabin pessary for different clinical situations

	Proximal inner diameter		*Distal outer diameter*		*Height*			
Clinical situation & results from TVS	*32 mm*	*35 mm*	*65 mm*	*70 mm*	*17 mm*	*21 mm*	*25 mm*	*30 mm*
Short cervix 2nd trimester								
Singleton								
No or Y-shaped funneling								
Nulliparous	√		√			√		
Parous	√			√		√		
V- or U-shaped funneling								
Nulliparous		√	√			√		
Parous		√		√		√		
Twins								
No funneling								
Nulliparous	√		√				√	
Parous	√			√			√	
V- or U-shaped funneling								
Nulliparous		√	√				√	
Parous		√		√			√	
Short cervix (e.g. after cone biopsy) 1st trimester								
Singleton								
Nulliparous	√		√		√			
Parous	√			√	√			
Twins								
Nulliparous	√		√			√		
Parous	√			√		√		
Additional signs of 'prolapse' in any patient								
Nulliparous		√	√					√
Parous		√		√				√

Definition of 'short cervix' is relative and centile values specific for gestational age and different populations are preferred for definition of cut-off values. TVS, transvaginal sonography.
