# Supplementary material for: Comparing cervical cerclage, pessary and vaginal progesterone for prevention of preterm birth in women with a short cervix (SuPPoRT): A multicentre randomised controlled trial
Source: PLoS Med. 2024 Jul 16;21(7):e1004427. doi: 10.1371/journal.pmed.1004427 (PMC11288449; doi:10.1371/journal.pmed.1004427)
Supplement: Attachment — Submitted filename: Response to reviewers NHNSASfinal.docx [file pmed.1004427.s011.docx]

Response to reviewers

1. Editor’s requests

a) Please adapt the title to PLOS Medicine style, and we suggest: "Comparing cervical cerclage, pessary and vaginal progesterone for prevention of preterm birth in women with a short cervix (SuPPoRT): A multi-centre randomised controlled trial".

**Response:**

**Title adapted as suggested**

b) We ask you to adapt the abstract to the three-part journal style. The final sentence of the 'Methods and findings' subsection should begin "Study limitations include ..." or similar and should quote 2-3 of the study's main limitations. Please mention stopping the trial in the abstract.

**Response:**

**Altered as suggested.**

c) Please substitute the 'Key points' with an 'Author summary' section in journal style (you may find it helpful to consult one or two recent PLOS Medicine research papers to get a sense of the preferred style).

**Response:**

**Included**

d) Rather than "In women with a short cervix, cerclage ... are equally efficacious ..." (as in the current abstract), we ask you to adapt the conclusions throughout to "In this study, we found that ... were equally efficacious ... as judged with a 20% equivalence margin." or similar. 

**Response:**

**Adapted as suggested.**

e) Please ensure that the Discussion section (main text) includes a paragraph on study limitations.

**Response: Included**

f) Please include a completed CONSORT checklist with your revision, labelled 'S1_CONSORT_Checklist' or similar and referred to as such in the text.

**Response: Included as supplementary Figure 1 and referenced in the text.**

g) In the checklist, please refer to individual items by section (e.g., "Methods") and paragraph number, not by line or page numbers as these generally change in the event of publication. 

Please also consider using the CONSERVE extension if appropriate.

**Response:**

**Altered as suggested**

Comments from the reviewers:

*** Reviewer #1: 

Alex McConnachie, Statistical Review

Hezelgrave et al present the results of a three-arm equivalence trial of treatments for the prevention of preterm birth in women with a short cervix. This review considers the statistical aspects of their paper.

I found this to be a well written and interesting paper. The very high follow-up rate is a strength, and to be commended. I have a number of comments about the analysis, though these should all be possible to address.

The sample size calculation is presented as it is described in the protocol, which was reviewed by the funders and approved by the regulators, and in that sense is correct. The fact that it is based on a two-group test of superiority, rather than equivalence, is unfortunate, though in fact makes little difference. By assuming that the average primary outcome rate to be 50%, the sample size calculation was actually conservative, and the sample size required would have been smaller for any other true average (as turned out to be the case).

Quite rightly for an equivalence study, the authors say that they consider differences in treatment effect in either direction, citing Jones (1996). However, Jones (1996) is quite clear in that the analysis should be based on confidence intervals. For any two treatments, equivalence can be declared if the confidence interval for the treatment effect difference lies entirely within the equivalence limits, in this case the range from -20% to +20%. The approach taken in this paper, of declaring equivalence if the analysis comparing all three treatments is not statistically significant at 5%, is not the same thing. For example, a very small study would be unlikely to show statistical significance between groups, yet the confidence intervals for differences between treatments would be wide, and likely not be contained within the equivalence limits. On the other hand, a very large study might be able to detect treatment effect differences, even if these differences are small, and
the treatments should be declared equivalent within the specified limits.

**Response:**

**We have made changes to the methods to reflect the above comment on equivalence**

I suggest that the overall test of differences between groups has no bearing on the equivalence or otherwise of the three treatments, and should not be the main focus of the analysis. The things that matter are the estimated differences between treatments, and their confidence intervals. If these intervals lie within the equivalence limits, then equivalence can be declared. According to Tables 2 and 3 in the paper, this appears to be the case (just), so it seems that the overall conclusion of the paper is preserved.

This applies also to the secondary outcomes. It is not OK to omit the between-group comparisons and confidence intervals, on the basis of a non-significant three-way comparison. Even though no equivalence limits were pre-specified (as far as I can see) for the secondary outcomes, as an equivalence study, the focus should remain on these treatment effect differences and their confidence intervals. Given the low event rates, these CIs will likely be wide, which limits what can be said about the equivalence, or otherwise, of the treatments, but that is to be expected for secondary outcomes.

**Response:**

**We accept the reviewer’s strictures and have added in the confidence intervals as asked. We have not changed our conclusions, as the secondary endpoints were not fully powered, and the CI are wide.**

Whether the width of the confidence intervals reported should have been adjusted for multiple comparisons is debateable, and given how close the confidence interval for the Arabin vs. Progesterone comparison comes to a 20% difference, this could perhaps be acknowledged in the discussion. I have to say that the justification for not applying an adjustment ("As all pairwise comparisons were of interest, a Bonferroni correction was not included") is not one I have heard before, and sounds wrong to me - surely the fact that all comparisons are of interest would support the need for adjustment?

**Response:**

**Thank you for your comment.**

**We have not found any papers that suggest adjusting p-values in multi-arm equivalence studies by Bonferroni or similar methods. Indeed, two papers (Chang 2014 and Tseng 2021) in discussing such trials explicitly mention p<0.05 and make no comment on the possibility of adjustment.**

**Such adjustment would widen the confidence intervals and so reduce the chance of finding a significant difference between treatments. As all comparisons between treatments are interest, we consider that such adjustment would be inappropriate.**

**References:**

**Yu-Wei Chang, Yi Tsong, Xiaoyu Dong & Zhigen Zhao (2014) Sample Size**

**Determination for a Three-Arm Equivalence Trial of Normally Distributed Responses, Journal of Biopharmaceutical Statistics, 24:6, 1190-1202,**

**DOI: 10.1080/10543406.2014.948552**

**https://doi.org/10.1080/10543406.2014.948552**

**Yi-Kuan Tseng & Ken-Ning Hsu (2021) Study design for a three-arm equivalence clinical trial with binomially distributed outcomes,**

**Journal of Biopharmaceutical Statistics, 31:6, 736-744, DOI: 10.1080/10543406.2021.1968891**

[**https://doi.org/10.1080/10543406.2021.1968891**](https://doi.org/10.1080/10543406.2021.1968891)

Personally, I think that the equivalence limits of +/- 20% are quite wide. The treatment effect for all three treatments was assumed to be a 25% absolute reduction in PTB (from 75% to 50%). However, this was pre-specified, and accepted by the regulators, so cannot be changed. Nevertheless, whether a 20% treatment effect difference is sufficiently small to be considered equivalent could perhaps be discussed.

**Response:**

**As the reviewer notes, equivalence at 20% difference was accepted by the funders, as part of the original protocol. We have added a comment to the discussion:**

***“In this pragmatic trial we show that there is no clear advantage between the three therapies to prevent PTB before 37 weeks’ gestation, as judged with a 20% equivalence margin.”***

The primary analysis, as prespecified in the analysis plan, was "intention-to-treat". Given that ITT analyses tend to bias towards the null, equivalence studies would normally use a per-protocol population for the primary analysis. Saying that, it is usually recommended to look at the analysis in multiple ways, and the authors include a PP analysis, according to their analysis plan, which is good. However, what they report appears to be more of an "as-treated" analysis, rather than "per-protocol", since the denominator in the progesterone group is larger in Table 3 than in Table 2. For me, "per-protocol" would involve the exclusion of anyone who did not receive their allocated treatment, or otherwise deviated from the protocol. An "as-treated" analysis is fine to do, but should be specified as such.

**Response:**

**We have changed the definition of our per protocol analysis to “as treated analysis” in the methods and have performed a PP analysis (to comply with the above definition) and have included this in the supplementary. There remains no significant difference between the 3 groups.**

Finally, there is a comment regarding the decision to end the study early which includes the text "the DMC (who reviewed the blinded data)". Usually, the DMC get to see unblinded data - is this a typo?

**Response:**

**The reviewer is correct. We have changed the text.**

*** Reviewer #2: 

1. The principal, obvious, problem, is its size and the ensuing the lack of statistical power. The design was for a primary outcome of birth <37 weeks; even if the numbers in the power calculation had been achieved, it would always have been under-powered for the more important secondary outcomes of birth <34 or 30 weeks, or severe morbidity. This was compounded by trial violations largely because of women's concerns. This issue needs to be better addressed in a limitations section, obvious though it is.

**Response:**

**Now added as a limitation to the discussion section:**

*“A further limitation was that the trial was stopped early due to the Covid-19 pandemic, although adequate power was achieved to evaluate the primary outcome as judged by the DMC. Finally, the study was underpowered for the arguably clinically more significant outcome of PTB prior to 30 and 34 weeks of gestation.”*

2. On design, the mixed risk groups make interpretation difficult. In real life, many women with a prior mid trimester birth or very preterm birth are started on progesterone, then serially scanned, and then have a cerclage if the cervix measures <25mm. Women with prior cervical surgery or an incidental finding of a short cervix do not receive progesterone until the cervix is found to be short. I do think the mix of 'risk' should be addressed in the limitations too.

**Response:**

**At the time of trial commencement, there was a paucity of evidence to support routine prescription of progesterone in women whose only risk factor is prior preterm birth, and in current UK clinical guidelines, advice is now to ‘consider’ use of vaginal progesterone’ in women with a prior history. Women were excluded from the SuPPORT trial if they had already been commenced on vaginal progesterone. The mix of risk is already addressed in the discussion as stated below:**

***“Given the breadth of risk factors for sPTB included in this pragmatic trial, it is possible that pooling patients limited the impact of treatments or masked differences between them”***

3. Although randomisation was properly conducted, it is noteworthy that among women allocated cerclage, there was a higher (not statistically significant) number of women both with a second trimester birth and a very short cervix (<15mm). A woman with a prior history and cervix <15mm is very high risk; one with previous cervical surgery and a cervix >15mm and <25mm is not.

**Response:**

**Many thanks for your comment. As you correctly state, these differences are not statistically different. The proportion of women with the shortest cervix <15mm was 12.5% (cerclage) vs 12.3 % (progesterone) vs 8.3% (pessary). Likewise for women with previous cervical surgery 42% vs 46% vs 49%. Given there was no apparent treatment effect when subgroup analysis for very short cervix and for risk factor (Supp Table 2) was carried out, then these are not likely to be confounders and we do not feel warrant further comment. We have added in a line in the text (results) to acknowledge this.**

***Of note, a slightly higher proportion of women in the cerclage and progesterone group had a cervix <15mm and a previous mid-trimester loss (Table 1), but these differences were not statistically significant”.***

4. Table 1: the column headings are confusing: '% (n/128) or mean (SD)'. The formatting needs improvement.
Table 2: the 'Components of the adverse perinatal outcome' are incorrectly aligned. The formatting needs improvement.

**Response:**

**Many thanks for pointing this out. They have now been improved for clarity : *N (%) or mean (SD), and formatting of table improved.***

5. The sentence '… we see ~40 in our large tertiary-referral preterm surveillance clinic per year…' seems out of place in a report of a multi-centre trial.

**Response:**

**We agree, now removed.**

Despite its small size, this trial, which must have been very difficult to conduct, is clearly worthy of publication in a journal such as Plos Medicine.

**Response:**

**Many thanks for your helpful comments.**

*** Reviewer #3: 

Introduction
1.    Short cervical length is typically considered less than or equal to 25mm

**Response:**

**The SuPPorT study used <25mm as a criteria for entry, consistent with the large cerclage RCTs (e.g Owen J, Hankins G, Iams JD, et al. Multicenter randomized trial of cerclage for preterm birth prevention in high-risk women with shortened midtrimester cervical length. *Am J Obstet Gynecol* 2009;201:375.e371-378.) . This is predefined in our protocol and doesn’t warrant changing now.**

2.    'Mid-trimester' should be defined with a gestational age range

**Response:**

**Now more clearly defined in manuscript**

3.    'No single-study' - would avoid making claims unless including information regarding a systematic review with search terms etc. that support this statement

**Response:**

**Amended to ‘the authors are not aware of any single study direct comparisons”. We feel that this statement is important to justification for current trial.**

Methods
1.    Should clearly state trial performed in singleton gestations only

**Response:**

**Now made clearer in introduction and methods.**

2.    Is short cervical length defined in this trial < 25mm or </=25mm?

**Response:**

**<25 mm (rationale as described above)**

3.    What was the rationale for including gestational age as early as 14 weeks if trial interventions have been typically studied starting at 16 weeks in prior randomized trials— is cervical length screening starting at 14 weeks standard of care in the UK?

**Response:**

**At the time of trial commencement, there was no ‘standard of care’ in the UK to define gestation at which cervical length scanning should start, and generally depends on the indication for the cervical screening. For example if women had prior mid-trimester loss at 15 weeks of gestation, it would be reasonable to start scanning at 14 weeks. 14 weeks was therefore chosen pragmatically not to miss this small proportion of women who may be referred early, and have a short cervix detected on ultrasound, although in practice, the majority of the women developed a short cervix (and were randomised at 19-20 weeks (Table 1).**

4.    For patients with a prior late miscarriage after 16 weeks of gestation, is a history-indicated cerclage not be routinely performed? Or is this in the presence of preterm labor symptoms (i.e. pain or bleeding)?

**Response:**

**As per UK NICE guidance (and existing trial evidence) there is no benefit to insertion of a cerclage in women with a history of mid trimester loss, nor is it indicated in the presence of symptoms.**

5.    Regarding procedures for cerclage- if a patient had a short cervix at 23+6 for example, they may have had a cerclage placed up to 7 days later?

**Response: Yes they may (consistent with UK guidelines). However, taking into account gestational age, if the stitch insertion was not possible within a reasonable timeframe or clinicians were not happy to randomize to the trial interventions, they were not included in the trial. In general, with advanced gestation, effort would be made to insert the stitch as soon as possible.**

6.    Did agents for tocolysis or antibiotics differ between centers?

**Response:**

**Routine use of indomethacin or other tocolytics at the time of cerclage insertion is not common practice in the UK, nor does it have good evidence, however it is used by some clinicians. We designed the SuPPORT trial to be pragmatic rather than prescriptive, collecting data on all adjuncts used. Indomethacin was only used on 7 stitch insertions, at 4 different hospitals. Prophylactic antibiotics were used similarly sparsely for 25 of the cerclage insertions, at 9 centres inconsistently. Given the small numbers, and lack of evidence for benefit for any of these adjuncts, we do not feel it is appropriate to factor these into our analysis.**

**For transparency, we have now included the following addition into the paper: “*Use of adjuncts at the time of stitch insertion were rare. Indomethacin was used in 7/111 (6.3%) of insertions, and prophylactic antibiotics were given for 25/111 (22.5%) of cases.”***

7.    Regarding procedures for vaginal progesterone- what was rationale for this dose and completion by 34 weeks instead of 36 weeks?

**Response:**

**We recommended vaginal progesterone use up until 34 weeks, as per standard NICE guidelines in the UK (consistent with routine clinical practice in most centres, and with the dose used by the large RCTs (e.g** <https://bmcpregnancychildbirth.biomedcentral.com/articles/10.1186/1471-2393-12-79>)

8.    Why were cervical lengths performed after cerclage placement?

**Response:**

**This is common practice in the UK and is known to be a good predictor of outcome (**[**https://obgyn.onlinelibrary.wiley.com/doi/10.1002/uog.26161**](https://obgyn.onlinelibrary.wiley.com/doi/10.1002/uog.26161)**). It allows decisions on future management and may change thresholds at which adjuncts such as steroids or in utero transfer are offered. Given that all three interventions are not perfect prophylaxis, it also allows early detecting of bulging membranes, when an emergency cerclage could be offered.**

9.    For the power analysis, none of the referenced studies had a PTB < 37 weeks rate as high as 75% in their respective control groups; please clarify

**Response:**

**The reviewer is correct that in the study populations in the cited trials, the control groups had lower preterm birth rates than our projected preterm birth rate. We thought it was more appropriate to look at the preterm birth rate in the potential (UK) population from which the trial would be recruiting. The available evidence tells us that there is an approximately 50% reduction in risk of preterm birth with use of the pessary, stitch and progesterone (references cited). Our previous experience (captured by a robust database of outcome data from >2000 women attending our prematurity clinic) indicates that approximately 50% of women with short cervices (<25 mm) treated with cerclage) deliver early <37 weeks. Thus we hypothesised that the preterm birth rate in our population would be closer to 75%. We have now made this more clear in the text.**

10.    First time mentioning fetal fibronectin is in the subgroup analyses; if this was routinely collected before each intervention suggest highlighting that in the procedures section of the methods

**Response:**

**Now highlighted in methods section**

Results
1.    The differences in maternal infection were highlighted by the authors in the results despite not reaching statistical significance—difference between progesterone group vs. pessary group was 7%; there was a 5% difference in adverse perinatal outcome between progesterone group and pessary group—why isn't that highlighted? Would suggest being consistent and stating 'similar' in results if there was no statistical significance and highlight any author interpretations in the discussion

**Response:**

**Now adapted as suggested.**

Discussion
1.    Suggest the authors comment on the following:
a.    Hypothesized mechanisms for differences in maternal infection pointed out among the 3 groups

**Response:**

**Now hypothesised in the discussion.**

b.    Evidence has supported the use of vaginal progesterone starting at 16 weeks for patients with a history of spontaneous preterm birth regardless of cervical length measurement; many centers are offering this routinely, how generalizable are these findings?

**Response:**

**At the time of trial conception, it was not routine practice to prescribe progesterone for all patients with a history of preterm birth. Furthermore, for all women with a history of preterm birth (regardless of cervical length) the most recent meta-analysis evaluating vaginal progesterone use in women with a history of preterm birth only, showed no benefit from vaginal progesterone (Conde-Agudelo A, Romero R. Does vaginal progesterone prevent recurrent preterm birth in women with a singleton gestation and a history of spontaneous preterm birth? Evidence from a systematic review and meta-analysis. Am J Obstet Gynecol. 2022 Sep;227(3):440-461.e2. doi: 10.1016/j.ajog.2022.04.023. Epub 2022 Apr 20. PMID: 35460628; PMCID: PMC9420758.).**

c.    If there is no clear advantage to pessary and evidence has demonstrated harm, why should people still consider this option?

**Response:**

**Added in a sentence: “*Thus, in the absence of more reassuring data, cerclage and progesterone would be the preferred interventions of choice*.”**

d.    Suggest stating strengths and limitations in dedicated paragraphs
-  patient demographics compared to previous studies
-  low number of patients with a very short cervical length <15mm

**Response:**

**Conclusion rearranged to reflect these comments. Also added: “*Numbers of women with very short cervixes <15mm were also too low to confidently assess treatment effect for the highest risk women*”.**

*** Reviewer #4: 

This is an RCT to evaluate the efficacy of pessary, cerclage and progesterone in the prevention of preterm birth in patients with a short cervix. 

The main concern of this RCT is the heterogeneity of the study population. The authors address this problem themselves on page 23: 'Our study, however, only included women with a short cervix; some of these women would have had a history of sPTB, but the majority did not (> 60%). Therefore, comparison with previous trials is difficult. Given the breadth of risk factors for sPTB included in this pragmatic trial, it is possible that pooling patients limited the impact of treatments or masked differences between them. As we learn more about the heterogeneous mechanisms of sPTB, future studies should explore sub-groups of women at high-risk. Given the recruitment challenges encountered by large individualized RCTs such as this one, novel research methodologies may be
required."

Other major concern is that 35 of the 385 patients (9,1%) did not receive the intervention they were randomised for. This is potentially prone to bias and patient selection. Since both patients and doctors may be more confident with one treatment as opposed to another based on patient characteristics and history. Moreover, in an additional 6,2% an emergency cerclage was inserted.

However, if consideration is given to clarify certain aspects this is an interesting trial. It is difficult to conduct trials like this and takes a lot of effort. And although (as the authors state themselves) it will be difficult to compare this trial with others, it does add additional information.

INTRODUCTION
*    "Thus, there is no consensus as to the optimal management of high-risk asymptomatic women who develop a sonographically short cervix."

I do not understand this sentence, it seems like a mixture of definitions, since all the women in this trial had a short cervix, but not every woman was defined as high risk prior to cervical length measurement. High risk was defined as: previous history of PTB or cervical surgery. 15% of the women in this trial became high risk because of a short cervix. Please rephrase.

**Response:**

**We appreciate the reviewers concern regarding this sentence. We feel that the adapted sentence “*Thus, there is no consensus as to the optimal management of high-risk asymptomatic women with a sonographically short cervix*” accurately defines the clinical dilemma that the trial seeks to address, without stating that the high risk nature of the pregnancy necessarily came BEFORE the cervix was measured.**

*    To my knowledge the risk of preterm birth is higher in case of a history of PTB plus a short cervix compared to surgical surgery plus short cervix compared to just an asymptomatic short cervix.  
The authors tried to correct for this risk difference by stratifying for different variables. The authors should elaborate on the rational for including different groups of patients in the discussion.

**Response:**

**The reviewer makes a good point, largely driven by the fact that for most of these interventions, we only have good evidence for effect in those women with a previous history of preterm birth. In a real-world setting, we are frequently confronted with women more moderate risk factors for a preterm birth with a short cervix, who may deliver prematurely. Thus we designed the trial to include all-comers with a short cervix. We have addressed this in the discussion when we write. *“Given the breadth of risk factors for sPTB included in this pragmatic trial, it is possible that pooling patients limited the impact of treatments or masked differences between them” .* It is indeed reassuring that in our sub-analysis there was no signal of difference between intervention in only those with previous preterm birth. We have elaborated on the discussion by adding; ‘*though sub-group analysis was reassuring’.***

METHODS
*    What is the definition of history of previous spontaneous preterm birth? < 34 weeks or < 37 weeks

**Response: clarified to <37 weeks of gestation**

*    Both pessary and cerclage were removed at 37 weeks', however progesterone only till 34 weeks'. Please explain the rational for this difference.

**Response:**

**We recommended vaginal progesterone use up until 34 weeks, as per standard NICE guidelines in the UK (consistent with routine clinical practice in most centres, and with the dose used by the large RCTs (e.g v** <https://bmcpregnancychildbirth.biomedcentral.com/articles/10.1186/1471-2393-12-79>)

*    How was the pessary size determined? And which sizes were used?

**Response:**

**We have added “*a suitably sized pessary “(as per manufacturer recommendations, guided by parity and presence of a funnel, either 32 x 65 x 21mm or 32 x 70 x 21mm)”.* The sizing table is included in the protocol (which has been published).**

*    Primary outcome was delivery < 37 weeks.

**Response:**

**This is correct**

*    On page 16 in the adverse events on safety outcomes, there suddenly appears information on the type of analysis: 'Data analysis was by intention to treat, however, proportion of women who did not receive the primary intervention, who stopped the primary intervention early, and/or received alternative treatment were also examined." Should this section not be moved to the Statistical Analysis section?

**Response:**

**Thank you for pointing this out, moved to a more appropriate sub-section.**

*    Moreover: what is the definition of early?

**Response :**

**Added ‘prior to protocol defined removal- which encompasses 34/37 weeks (progesterone/cerclage and pessary) as well as medically indicated removal for PPROM/TPTL.**

*    What is the definition of the per protocol analysis? This is not clearly stated in the paper. In the SAP it reads:

"A secondary analysis of the 'per protocol ' (PP) population will also be carried out: limited to participants who were randomised, and who received only one intervention, according to the intervention administered, even if it was not the intended one. Women who received more than one intervention or none at all will be excluded from this analysis. Rescue cerclage for bulging fetal membranes will not be considered an additional intervention for this analysis however, as this is a secondary end-point in itself, and is not considered equivalent to cervical cerclage for a short cervix."

Although the authors do provide a per protocol analysis it is unclear how the study population in this analysis was defined, since also the SAP is not clear enough. What about women who only used progesterone for 2 days, or 14 days? Do we have any data on compliance? Same for pessary. I do not understand why women with an emergency cerclage were included in the per protocol analysis, since it might influence your primary outcome.

**Response:**

**We have also performed a per-protocol analysis (Supplementary Table 4) which shows similar results to the intention to treat and as-treated analysis. However, we have not removed the patients who required an emergency/rescue cerclage from this analysis as this was a secondary outcome in our study protocol and therefore, not a protocol deviation (Hezelgrave et al., 2016; DOI: 10.1186/s12884-016-1148-9).**

*    I do not completely understand the Sample Size calculation. to reduce the rate of PTB < 37 weeks in high-risk women with a short cervix from approximately 75% (untreated) to around 50% (refs 8, 12,13). In Goya the reduction of PTB < 37 weeks is from 59% to 22% (pessary). In Owen from 60% to 45% (cerclage) and Romero from 37 and 43% (progesterone).

**Response:**

**This has now been updated to be more clear, as per the above reviewer’s similar comment.**

*    In the subgroup analyses it is mentioned that cervicovaginal fetal fibronectin concentration > 200 ng/ml pre-intervention was measured. This is also documented in the SAP, but the work-up of patients is nowhere mentioned in this paper.

**Response:**

**Now added to methods section**

*    There is a subgroup analysis for previous spontaneous PTB (in supplemental table 2). I do miss the subgroup analysis for cervical surgery. Stratification was performed for both variable. Please add.

**Response:**

**Now added to Supplementary table, results for women without a history of preterm loss (the majority of whom had cervical surgery) as per the pre-defined sub-analysis**

RESULTS
*    This was based on having recruited > 75% of the intended 510 participants, and that the achieved sample size of 386 gave 85% power to achieve the same agreement as originally planned within 20% equivalence.
I do not understand this statement, please check.

**Response:**

**This statement has been checked by our statistician and is valid. 386 women gave us 85% power to define equivalence (agreement within 20%). Now slightly altered for clarity to:**

**“*Due to an externally imposed halt in recruitment over the COVID-19 pandemic, the sample size was reviewed and on the advice of the Independent TSC, and the DMC (who reviewed the blinded data), the trial was stopped. This was based on having recruited > 75% of the intended 510 participants, and that the achieved sample size of 386 gave 85% power to define equivalence as originally planned (agreement within 20%).”***

*    The authors state that "a higher rate of maternal infection at delivery was noted in the progesterone arm (21.5%) vs 12.7% in the cerclage and 14.0% in the pessary groups, but this did not reach statistical significance (Table 2). "
This finding is not further addressed in the discussion as to the cause of this difference

**Response:**

**Now hypothesised in the discussion**

*    There are 385 women in the ITT and 382 in the per protocol analysis: I do not understand this small difference! 
How were the patients assigned to the different groups in the per protocol analysis?

**Response:**

**We have included an as-treated analysis (original per-protocol analysis) and a per-protocol analysis and have clarified the definitions of these in the methods.**

22 women randomized to cerclage did not have it placed due to either clinician inability to place the cerclage at time of insertion (n=6), or patient request (n=16). Of these, 21 women received progesterone as an alternative treatment, and one received a cervical pessary.
Potentially this may influence your per protocol analysis especially since patient choice was the main factor in 16 women (plus in 7 in the pessary group and 6 in the progesterone group). Should these patients not be removed from the per protocol? 
Were these all lower risk women? For instance with a previous term birth? Or high risk women with an extreme preterm birth requesting a surgical intervention. What are the baseline characteristics of these women, since these may very well influence patient and doctors distress as mentioned in the discussion.

**Response:**

**We have now done an additional pre-protocol analysis that has excluded these and found no difference, see above.**

Please adjust Figure 1 it states: the number of women receiving a different treatment do not match with the text 24 vs 22 (cerclage), 6 vs 7 (pessary)  and 4 vs 6 (progesterone).

**Response:**

**We apologise for the confusion. Figure 1 is correct, we have modified the text.**

How could centres participate in this trial if the clinician could not perform a cerclage?

**Response:**

**The clinician inability to place cerclage refers to the perceived insufficiency of the cervix, rather than clinician skill. We believe this is clear by emphasising ‘*at the time of insertion’***

Additionally (page 20) in the pessary group the device was removed in 13 patients and 7 women requested alternative treatment prior to insertion (patient choice). 
In the progesterone group: 11 women receive a cerclage: 6 at patient request. 
Moreover, it states that emergency sutures for bulging fetal membranes were inserted for 14 women (10 women randomised to progesterone and 14 randomised to cervical pessary). Should this be 24 patient (6,2%)? And as already mentioned above: I think these patients should also be removed from the per protocol analysis.

**Response:**

**We apologise for the mistake and have updated the figures for emergency sutures in the text – there were 14 emergency sutures (9 randomised to progesterone and 5 randomised to pessary).**

**We have also performed a per-protocol analysis (Supplementary Table 4) which shows similar results to the intention to treat and as-treated analysis. However, we have not removed the patients who required an emergency/rescue cerclage from this analysis as this was a secondary outcome in our study protocol and therefore, not a protocol deviation (Hezelgrave et al., 2016; DOI: 10.1186/s12884-016-1148-9).**

*    The PTB rate was 108/381. However there are 385 patients. In the text it is stated that only 4 women had an indicated PTB is that correct? – clarify loss to follow up (4)
104/385 spontaneous preterm births and only 4/385 iatrogenic? AND 108 /385 overall PTB. Please make clear whether you are discussing overall preterm birth or spontaneous preterm birth throughout the paper? 
Does the same apply to (spontaneous) PTB < 30 and 34 weeks'?

**Response: 386 women were randomized. 5 were lost to follow up, leaving 381. The 4 women with an iatrogenic preterm birth are included in the 381.**

**The primary and secondary analyses were preterm birth (spontaneous and indicated) <37, 34 and 30 weeks. Now clarified in the ‘outcomes’ section of the methods section, and is clear in the consort diagram.**

DISCUSSION
*    The discussion is well written and nice to read. All relevant references are included. 

*    I do wonder about the statement on page 21: 'Commencing any of these
therapies as first line treatment of a short cervix would be reasonable,
within a shared decision-making model", since almost 10% of the patients in this trial did not receive the allocated treatment. And another 11% changed treatment. Please comment on that.

**Response: The reviewer is correct to point out that in a real-world setting, patients and clinicians have treatment preference, and intolerances to treatment, meaning that even in. a trial setting (and more so in rea life) there is fluidity to treatment decisions. The trial is a pragmatic one, and reflects real world treatment dilemmas. It shows that it is reasonable to commence the therapies, but that there may be fluidity in treatment due to patient and clinician preference.**

*    It is interesting to read that only 19% of UK PTB clinics use progesterone in patients with a short cervix. There is no mention of the use of progesterone in patients with a previous preterm birth? Is this also not implemented in the UK? From how I read this paper, progesterone was not routinely used in patients with a previous preterm birth in this study (expect for the 4 in the cerclage group as mentioned). Maybe this should be added in the methods and added in the discussion.

**Response: At the time of trial commencement it was not routine to prescribe progesterone for women with a history of preterm birth, as discussed above. The methods emphasise the exclusion of these women ‘*Women were excluded if they had persistent vaginal bleeding, visible membranes on speculum, severe abdominal pain, sepsis, PPROM and contraindications to, or pre-existing use of the study interventions*’. Now added to the discussion: “and at the time of trial commencement, evidence for progesterone treatment for all women with a history of preterm birth was lacking”.**

Table 1. 
*    Baseline characteristics: the phrases previous late miscarriage and midtrimester loss are used in Table 1. Please use one of those.

**Response: adapted as suggested**

*    There seem less nulliparous women in the progesterone group. 56% in progesterone vs 68% in Arabin vs 67.7% in cerclage. Does this affect the results? Please comment on this in discussion

**Response: When considering risk, the more appropriate consideration is those with previous pregnancies 14 weeks and over (as previous early miscarriages likely do not affect preterm birth risk), and these numbers are stated in the row below. This difference is not statistically significant (p=0.08). Differences in multips and primips with a short cervix is not known to be a substantial confounder (excluding those with previous preterm birth vs not, which is addressed in the subanalysis) and therefore unlikely to affect results. Non-significant variation in some variables is inevitable with a large RCT such as this.**

*    Please add mean GA of the detection of a short cervix.

**Response: we have included the mean age of gestation of intervention- the gestational age of detection will be -0-7 days of this, and we do not feel that this needs additional calculation.**

Table 2:
*    Please show data for PTB and spontaneous PTB separately.

**Response: In this group, there were only 4 iatrogenic births. We therefore do not feel it is necessary to dissociate this data, and the protocol defined primary analysis was preterm birth (not spontaneous preterm birth).**

Is there a difference between the time of detection of a short cervix and onset of intervention between the groups?

**Response: There will be inevitably be a small delay in arranging cerclage as this is an operative procedure- this is built in as part of the intervention and we do not feel it is meaningful to analyse this separately.**

Figure 1:
*    Please make sure the numbers in the figure and the paper match.
**Response: This has now been checked, and match**
